# TGFβ-mediated MMP13 secretion drives myoepithelial cell dependent breast cancer progression

**DOI:** 10.1038/s41523-023-00513-6

**Published:** 2023-03-02

**Authors:** Shayin V. Gibson, Elena Tomas Bort, Lucía Rodríguez-Fernández, Michael D. Allen, Jennifer J. Gomm, Iain Goulding, Ulrich auf dem Keller, Andrea Agnoletto, Cathrin Brisken, Barrie Peck, Angus J. Cameron, John F. Marshall, J. Louise Jones, Edward P. Carter, Richard P. Grose

**Affiliations:** 1 Centre for Tumour Biology, Barts Cancer Institute, Queen Mary University of London, London, EC1M 6BQ UK; 2 Department of Biotechnology and Biomedicine, Technical University of Denmark, Kgs. Lyngby, Denmark; 3 ISREC – Swiss Institute for Experimental Cancer Research, School of Life Sciences, Ecole polytechnique fédérale de Lausanne (EPFL), SV2.832 Station 19, 1015 Lausanne, Switzerland

**Keywords:** Breast cancer, Extracellular matrix

## Abstract

Ductal carcinoma in situ (DCIS) is a non-obligate precursor of invasive breast cancer. Virtually all women with DCIS are treated, despite evidence suggesting up to half would remain with stable, non-threatening, disease. Overtreatment thus presents a pressing issue in DCIS management. To understand the role of the normally tumour suppressive myoepithelial cell in disease progression we present a 3D in vitro model incorporating both luminal and myoepithelial cells in physiomimetic conditions. We demonstrate that DCIS-associated myoepithelial cells promote striking myoepithelial-led invasion of luminal cells, mediated by the collagenase MMP13 through a non-canonical TGFβ – EP300 pathway. In vivo, MMP13 expression is associated with stromal invasion in a murine model of DCIS progression and is elevated in myoepithelial cells of clinical high-grade DCIS cases. Our data identify a key role for myoepithelial-derived MMP13 in facilitating DCIS progression and point the way towards a robust marker for risk stratification in DCIS patients.

## Introduction

The introduction of mammographic screening programmes has resulted in the increased reported incidence of ductal carcinoma in situ (DCIS), with diagnosed cases increasing 33% over the last decade^1^. Although DCIS is a precursor lesion, studies suggest that approximately 50% of diagnosed DCIS will not progress to invasive ductal carcinoma (IDC)^2,3^. Despite this, the standard treatment for 99% of DCIS patients consists of either local wide surgical excision or mastectomy. However, regardless of treatment, the risk that these patients will die from their disease is less than 2–3%^4,5^. Thus, there has been growing concern regarding the over diagnosis and overtreatment of many women^2,6,7^.

Previous efforts to identify predictive biomarkers have focused on comparing tumour cells in DCIS with those of IDC^8–11^. These studies have yielded limited information, reporting negligible differences in the signature of cancer cells throughout disease progression. In situ gene expression profiling of the malignant epithelial compartment, revealed extensive similarities in the transcriptomic signature between premalignant, preinvasive and invasive disease^9^, raising the question of whether the most critical changes that underpin disease progression lie in the surrounding microenvironment^12^. Indeed, analysis of transcriptomic changes across different cell compartments within the DCIS microenvironment reveal that compared to their normal counterparts, DCIS-associated myoepithelial cells show the most significant changes^13,14^.

In the healthy breast duct, myoepithelial cells provide the interface between luminal cells and the stroma, securing anchorage to the basement membrane via hemidesmosomes, and serve to propel milk through the ductal tree via contraction^15^. Healthy myoepithelial cells display tumour-suppressive roles, most likely through their expression of tumour suppressor proteins Maspin, P63 and P73^16–21^. Given their loss in IDC, the myoepithelial barrier has been long thought to act as a physical barrier, preventing carcinoma cells from invading into the stroma. However, emerging evidence suggests that myoepithelial cells can become altered to lose their tumour suppressive properties, and even enhance the proliferation and invasion of cancer cells^13,22–25^. Serial analysis of myoepithelial gene expression revealed that chemokines CXCL12 and CXLC14 were overexpressed in DCIS, with subsequent functional assays confirming their ability to enhance proliferation and migration of epithelial cells^13^. DCIS-derived myoepithelial cells also exhibit reduced Laminin-1 expression, which affects their ability to correctly polarise luminal cells^25^. Interestingly, spatial analysis of tissue from the Washington University Resource Archival Human Breast Tissue (RAHBT) cohort, which consists of matched primary DCIS tumours from women who later showed recurrent DCIS with invasive disease, revealed that myoepithelial loss was more pronounced in non-progressive cases. While a thin, discontinuous myoepithelium was present in the DCIS tumours of non-progressive patients, samples with more continuous myoepithelium were at higher risk of invasive recurrence^26^. Despite the correlation between myoepithelial integrity and invasive recurrence, whether myoepithelial cells can drive DCIS progression is yet to be elucidated^26^.

Here, we show that overexpression of myoepithelial β6 integrin, a marker of high-risk DCIS^24,27^, in a heterocellular spheroid model of DCIS, drives MMP13 dependent myoepithelial-led invasion of luminal cells. We demonstrate that this mechanism is facilitated via TGFβ-dependent activation of EP300, which epigenetically regulates expression of myoepithelial-derived MMP13. We postulate that this mechanism represents a key stage in DCIS, contributing to the growing evidence that myoepithelial dysfunction may act as a driver of early stage breast cancer progression.

## Results

### Overexpression of β6 integrin in myoepithelial cells drives myoepithelial-led invasion in spheroid model of DCIS

**Fig. 1:**
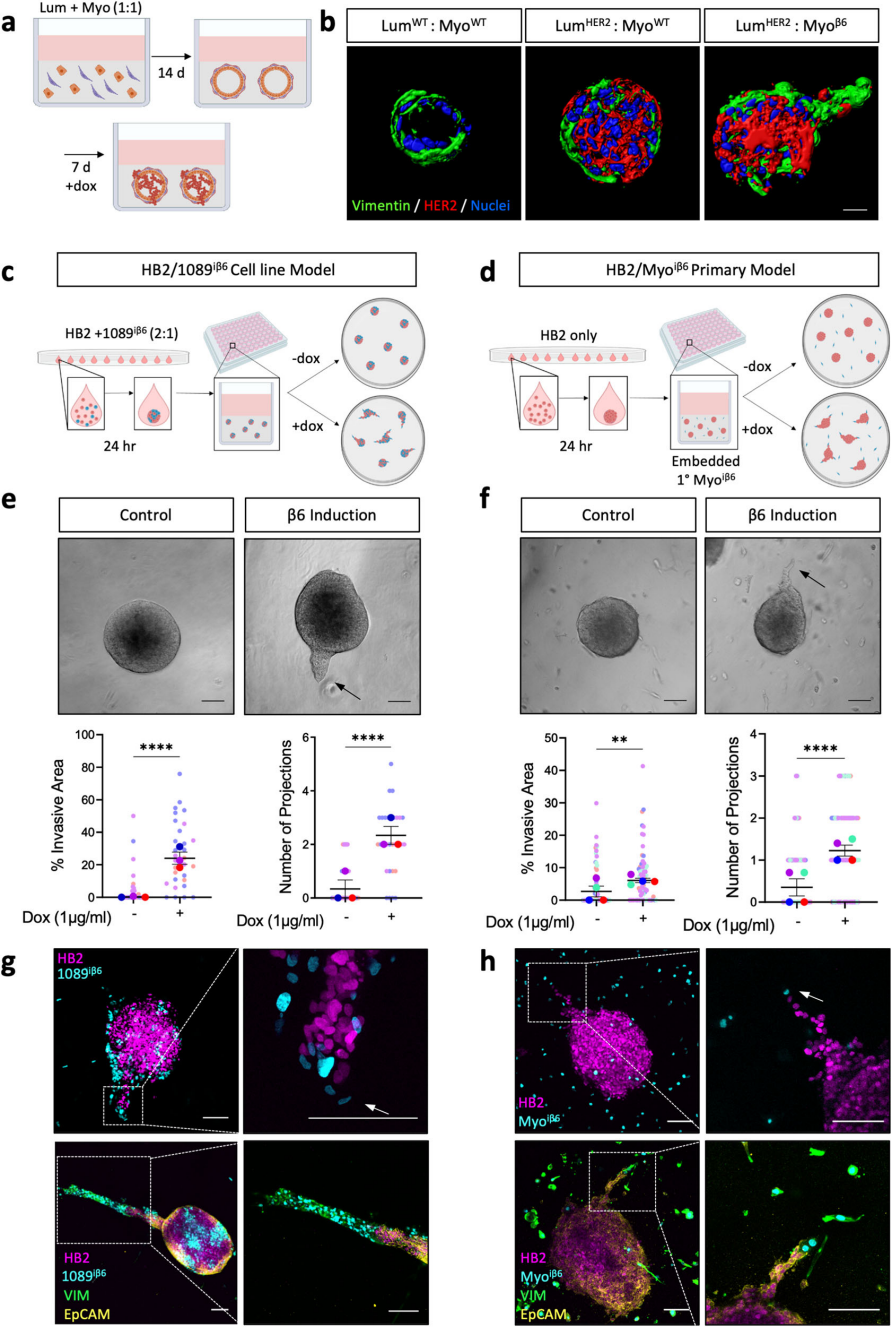
β6 integrin expression drives myoepithelial-led invasion in 3D. **a** Schematic of primary ductal model. Primary luminal and myoepithelial cells are embedded into collagen gels. Ductal structures are formed after 14 days and subsequently treated with doxycycline (1 µg/mL) for a further 7 days to induce transgene expression. **b** Imaris projections of HER2 (red) and Vimentin (green) expression in luminal/myoepithelial ductal structures after 21 days of culture with HER2 luminal expression −/+ myoepithelial β6 expression. Scale bar = 20 µm. **c** Schematic of spheroid model using HB2 and 1089^iβ6^ cell lines, where HB2 and 1089^iβ6^ heterocellular spheroids are embedded into 4 mg/mL collagen gels. **d** Schematic of spheroid model using HB2 luminal and primary myoepithelial cells where HB2 monoculture spheres are embedded into Myo^iβ6^-containing gels. Spheres are treated with doxycycline (1 µg/mL) to induce myoepithelial β6 integrin expression in both models. **e**, **f** Representative bright field images at day 4 −/+ doxycycline treatment. Summary graphs showing % invasive area and number of projections in (**e**) cell line and (**f**) primary myoepithelial cell models. Data are presented as mean ± SEM where each dot represents one sphere with biological replicates indicated by different colours. Average of biological replicates indicated as larger-sized points. **p < 0.01, ****p < 0.0001 (Mann–Whitney *U* Test). **g–h** Representative fluorescence images of invading spheroids with HB2 (magenta), 1089^iβ6^/Myo^iβ6^(cyan), Vimentin (green) and EpCAM (yellow) in (**g**) HB2/1089^iβ6^ and (**h**) HB2/Myo^iβ6^ models. Data are representative of at least three independent experiments. Scale bar = 100 µm.

Integrins are heterodimeric trans-membrane receptors comprising alpha and beta subunits that facilitate signalling between the intracellular and extracellular environments^28^. Integrin αvβ6 is an epithelial-specific integrin which is highly upregulated during wound healing and cancer^29,30^. During DCIS, mechanical stretching of the ducts drives myoepithelial expression of the β6 integrin subunit (β6)^27^, where its expression correlates with disease recurrence and invasive disease^24^. To investigate the biological consequences of myoepithelial β6 expression in vitro, we cloned the *ITGB6* open reading frame into a doxycycline-inducible lentiviral construct and generated both a β6-inducible immortalised 1089 myoepithelial cell line (1089^iβ6^) and primary, reduction mammoplasty-derived, myoepithelial cells (Myo^iβ6^) (Supplementary Fig. 1a, b). We have demonstrated that primary cultures of luminal and myoepithelial cells can be recombined in Collagen gels to form native ductal structures with a correctly orientated bilayer^31^. Induction of HER2 in the luminal compartment of this model, once ductal structures have formed, leads to luminal filling reminiscent of DCIS. Strikingly, combined expression of β6 in the myoepithelial compartment promotes the formation of invasive projections with leading myoepithelial cells and following HER2-positive luminal cells (Fig. 1a, b).

While our ductal model is a faithful reconstruction of the ductal architecture in an in vitro environment, the model is difficult to quantify and requires lengthy culture time that limits mechanistic studies. As an alternative, short term 3D co-culture systems were utilised, combining either 1089^iβ6^ or Myo^iβ6^ myoepithelial cells with the non-tumorigenic HB2 cell line^32^ into Collagen-I gels. For the 1089 myoepithelial cell line model, 1089^iβ6^ cells were combined with HB2 cells in hanging drops to form heterocellular spheroids, which were then embedded into Collagen-I gels (Fig. 1c). However, as primary Myo^iβ6^ cells collected in the centre of the sphere when combined with HB2 cells in hanging drops, and were then trapped, the primary myoepithelial cell model instead comprised of HB2 monoculture spheroids embedded in collagen gels containing distributed primary Myo^iβ6^ cells (Fig. 1d). Upon induction of myoepithelial β6 expression, we observed striking invasive projections across both models (Fig. 1e, f, Supplementary Fig. 1c, d). Sphere quantification and morphometric analysis revealed a significant increase in the percentage of invasive area, the number of projections and the projection length across both models following β6 induction (Fig. 1e, f, Supplementary Fig. 1c, d). Non-β6-induced control spheroids instead maintained their spherical shape, highlighting the requirement for myoepithelial β6 to drive the invasive phenotype (Fig. 1e, f, Supplementary Fig. 1c, d). To ensure that increased invasion was not a result of β6 induced proliferation, growth assays were performed, revealing no change in confluency or cell count following β6 induction in either cellular compartment (Supplementary Fig. 1e). Confocal analysis of fluorescently labelled cells demonstrated that while both myoepithelial and luminal compartments were present in the invasive projections, the invasion was in fact myoepithelial-led (Fig. 1g, h). Furthermore, while invading myoepithelial cells were Vimentin positive, luminal cells maintained expression of the epithelial specific marker EpCAM, demonstrating collective invasion and maintenance of the epithelial phenotype observed in tumour invasion (Fig. 1g, h)^33–36^.

### Myoepithelial β6 expression promotes protease dependent invasion through basement membrane remodelling

**Fig. 2:**
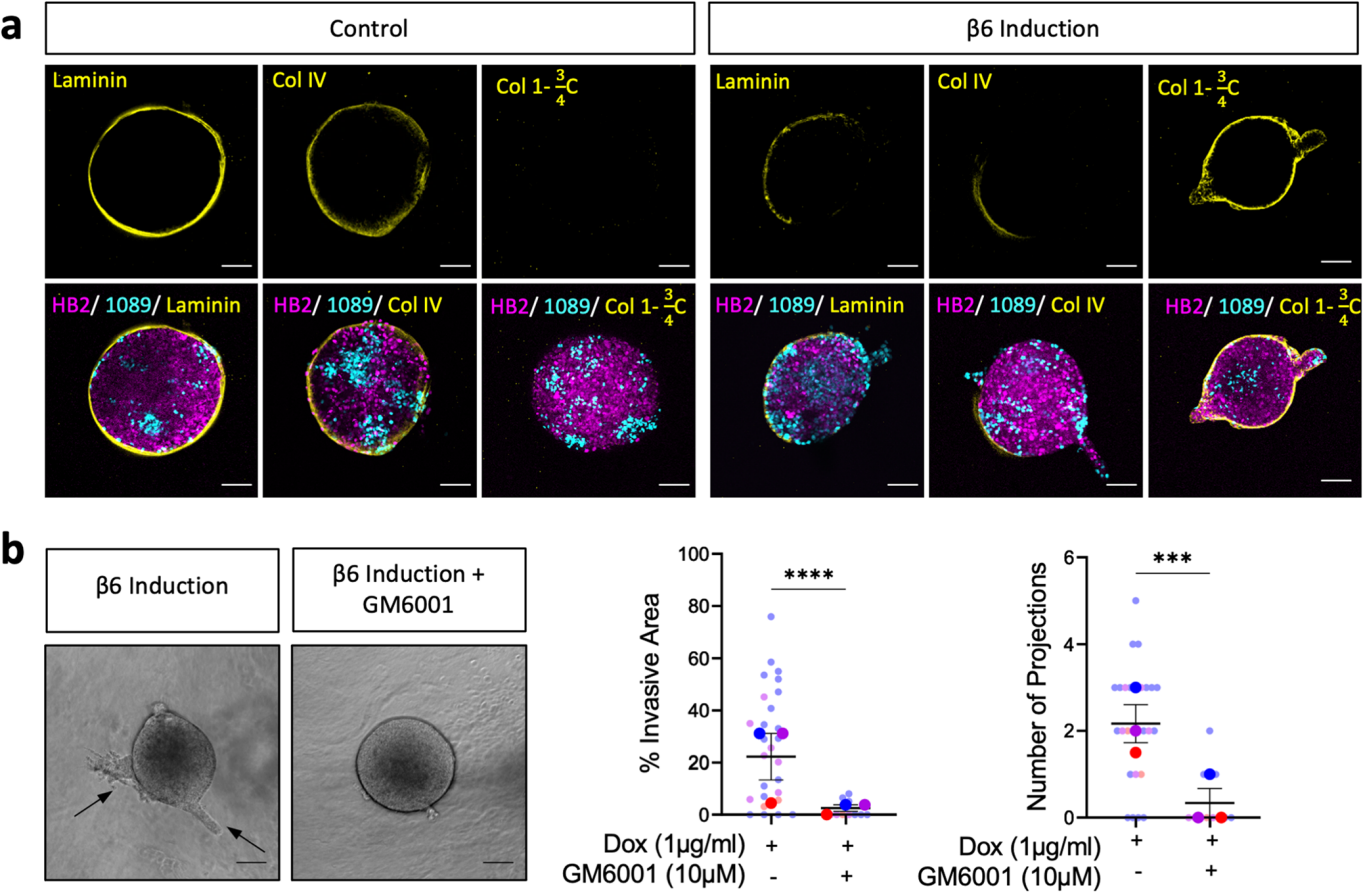
β6 driven myoepithelial-led invasion requires protease dependent cleavage of basement membrane proteins. **a** Representative fluorescence confocal sections of HB2/1089^iβ6^ spheres with HB2 (magenta), 1089^iβ6^ (cyan), and Laminin, Collagen IV or Cleaved Collagen I (yellow) in control and β6-induced conditions. **b** Representative bright field images of HB2/1089^iβ6^ spheres treated with doxycycline (1 µg/mL) and GM6001 (10 µM) at day 4 post treatment. Summary graphs showing percentage invasive area and number of projections. Data are presented as mean ± SEM where each dot represents one sphere with biological replicates indicated by different colours. Average of biological replicates indicated as larger-sized points. Data are representative of three independent experiments. ***p < 0.001, ****p < 0.0001 (Mann–Whitney *U* Test). Scale bar = 100 µm.

To determine whether spheroids deposited their own basement membrane (BM) and if this was perturbed by myoepithelial β6 expression, confocal imaging was used to visualise the major BM proteins, Laminin Alpha-I and Collagen IV. Reflective of an intact and deposited BM, continuous expression of both Laminin Alpha-I and Collagen IV was observed in equatorial sections of non-invading control spheroids. Despite minimal changes to matrix mRNA expression upon β6 integrin expression (Supplementary Fig. 2a), β6 myoepithelial-induced invading spheroids displayed a loss in deposited Laminin Alpha-I and Collagen IV, congruent with the loss of BM typically seen in the transition to IDC (Fig. 2a). Moreover, a striking increase in cleaved Collagen-I was detected in the gels of β6-induced spheroids compared to non-invasive controls, suggesting that while control spheres undergo minimal matrix proteolysis, this is enhanced upon expression of β6 in the myoepithelial compartment (Fig. 2a).

Stromal-led invasion can be protease dependent or independent, depending on cell behaviour and matrix composition^37–39^. We hypothesised that based on the expected pore size of the rat-tail derived Collagen-I used in our Collagen gels, as well as the increase in cleaved Collagen-I staining, that the observed myoepithelial β6-induced invasion was protease dependent^40^. To confirm this, spheroids were treated with a broad-spectrum metzincin inhibitor, GM6001 (10 μM), which targets several matrixin (MMPs) and adamalysin (ADAMs and ADAMTS) zinc endopeptidases. The percentage of invasive area, number of projections and projection length were significantly reduced following GM6001 treatment in β6-induced spheres, demonstrating that inhibition of metzincins is sufficient to block β6-dependent invasion in this model (Fig. 2b, Supplementary Fig. 2b). Indeed, analysis of Collagen-I cleavage showed a loss of β6-induced proteolysis following GM6001 treatment (Supplementary Fig. 2c). Furthermore, changes to the central area of GM6001-treated HB2/1089^iβ6^ spheres were non-significant, indicating that reduced invasion was not due to drug toxicity impacting cell growth (Supplementary Fig. 2b). Reduced invasion following GM6001 treatment was also recapitulated in the Myo^iβ6^ model, highlighting the requirement for a protease-mediated mechanism in facilitating invasion across both cell line and primary myoepithelial models (Supplementary Fig. 2d).

### β6-driven myoepithelial-led invasion is facilitated by MMP13 upregulation

**Fig. 3:**
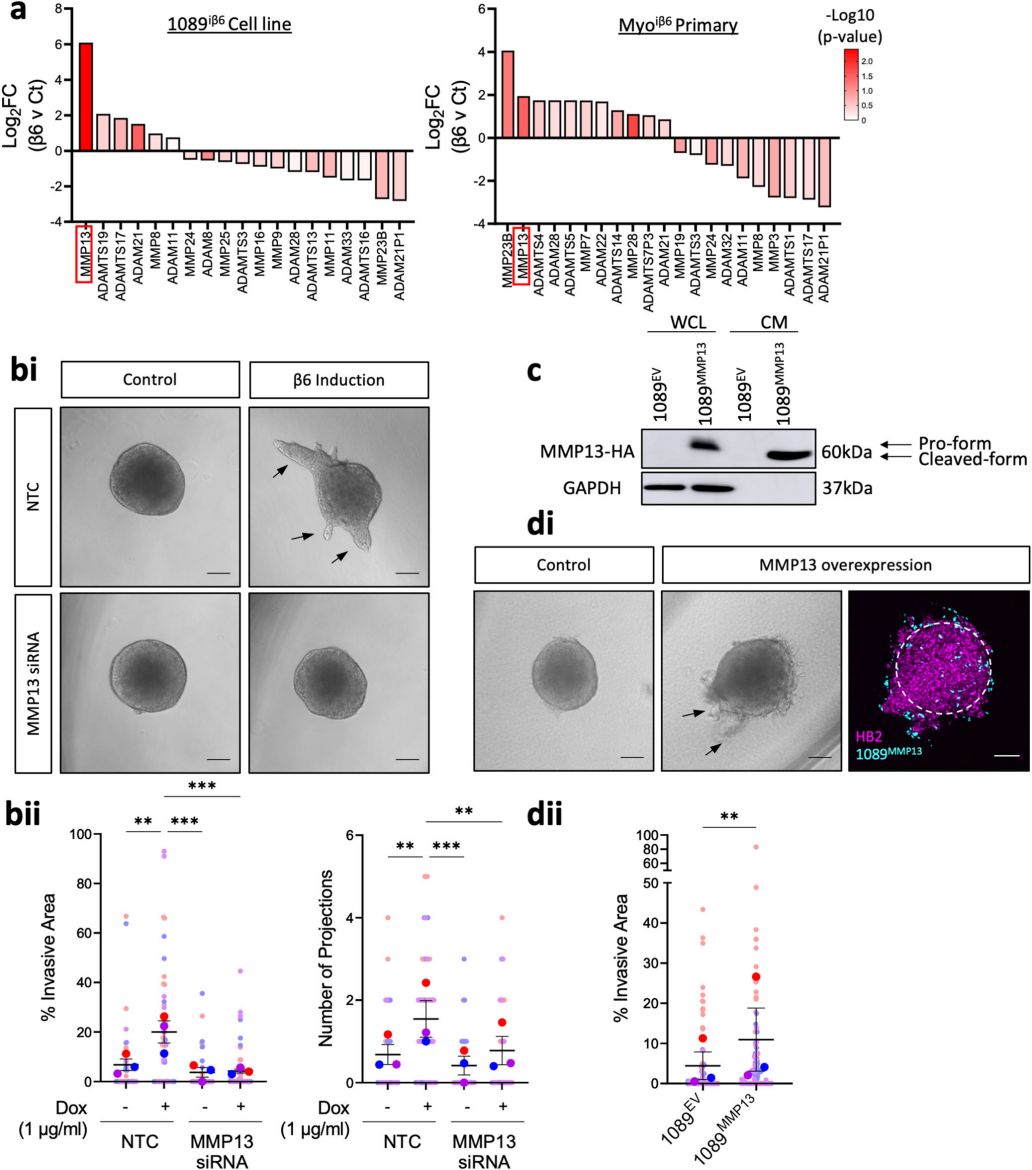
β6 drives upregulation of MMP13, which facilitates invasion. **a** Changes in protease mRNA expression following β6 induction in 1089^iβ6^ cell line (left) and Myo^iβ6^ primary cells (right). Data are representative of two independent experiments. **bi** Representative light micrographs of HB2/1089^iβ6^ spheres transfected with either non-targeting control (NTC) or MMP13 siRNA 4 days post doxycycline (1 µg/mL) treatment. **bii** Summary graphs showing percentage of invasive area and number of projections across conditions. **c** Western blot of MMP13-HA tag in whole cell lysate (WCL) and conditioned media (CM) of 1089 cells transfected with MMP13-HA pLenti-construct (1089^MMP13^) or empty vector (1089^EV^). **di** Representative light micrographs and (**dii**) summary graph showing percentage of invasive area and number of projections in HB2/1089^MMP13^ spheres. Data are presented as mean ± SEM, where each dot represents one sphere with biological replicates indicated by different colours. Average of biological replicates indicated as larger-sized points. **p < 0.01, ***p < 0.001 (Kruskal–Wallis Test with multiple comparisons or Mann–Whitney *U* Test). Data are representative of three independent experiments. Scale bar = 100 µm.

As broad metzincin inhibition was sufficient to block β6-dependent invasion, we next investigated changes to metzincin expression at the transcriptional level, following β6 induction in both 1089^iβ6^ and Myo^iβ6^ cells grown in 2D. Differential expression analysis of RNAseq data revealed changes in several metzincins, with the most significant change being an upregulation of MMP13 in both cell line and primary cell transcriptomes (Fig. 3a). To determine whether β6-driven invasion was facilitated by MMP13, we next examined whether loss of myoepithelial MMP13 via siRNA-mediated knockdown was sufficient to reduce invasion. Effective knockdown of myoepithelial MMP13 was confirmed prior to embedding spheres into Collagen gels (Supplementary Fig. 3a). Knockdown of myoepithelial MMP13 diminished invasion significantly compared to non-targeting control (NTC) conditions, implicating MMP13 as an active driver of invasion (Fig. 3b, Supplementary Fig. 3b). To confirm that this invasive phenotype also required proteolytic activity of MMP13, β6-induced spheroids were treated with an MMP13 specific inhibitor (CAS-544678 85-5; 1 µM)^41^. The percentage of invasive area, number of projections and the projection length were reduced significantly across both models, demonstrating that MMP13 expression and activity are both required to facilitate invasion (Supplementary Fig. 3c, d). This was coupled with immunofluorescent analyses of Collagen-I proteolysis, which revealed reduced cleavage of Collagen-I upon MMP13 inhibition in the HB2/1089^iβ6^ model (Supplementary Fig. 3e).

To determine whether overexpression of MMP13 could drive invasion independently of β6, we engineered a constitutive MMP13 HA-tagged expression lentiviral construct and transduced parental 1089 cells (1089^MMP13^). MMP13 overexpression was confirmed by western blot, with a significant increase in MMP13-HA detected in both the whole cell lysate and conditioned medium, compared to the 1089 empty vector control (1089^EV^) (Fig. 3c). Strikingly, overexpression of MMP13 was able to drive invasion compared to the empty vector (EV) control, with a significant increase in the percentage of invasive area (Fig. 3d, Supplementary Fig. 3f). Noticeably, 1089^MMP13^ cells exhibited more dispersed invasion out from the core sphere (Fig. 3d), compared to β6-induced spheroids, likely due to higher overall expression of MMP13. Taken together, this supports the role of myoepithelial-derived MMP13 as an active driver of β6-dependent myoepithelial-led invasion.

### MMP13 upregulation in β6-expressing myoepithelial cells is dependent on TGFβ signalling

**Fig. 4:**
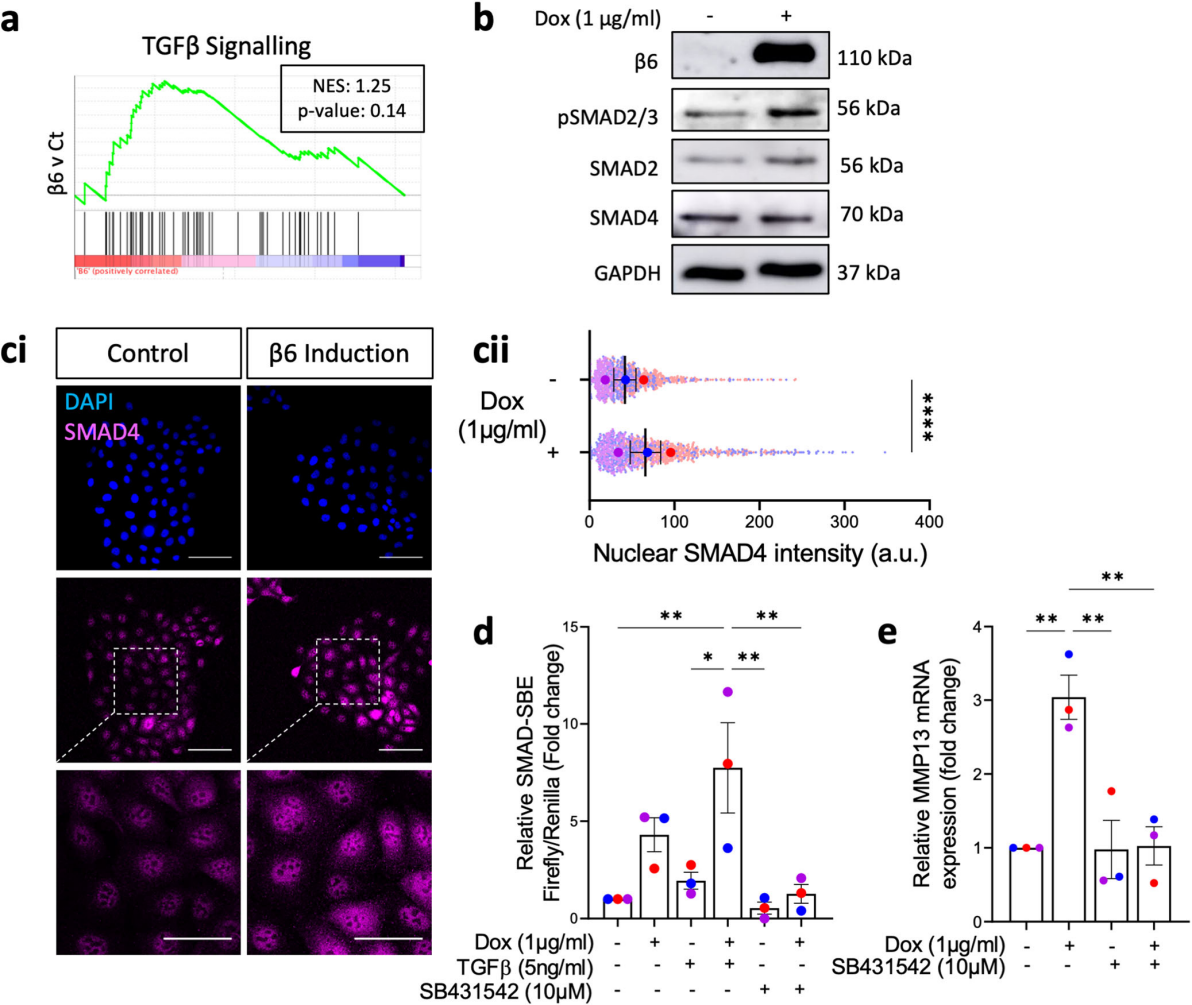
β6-driven MMP13 upregulation is dependent on TGFβ signalling. **a** GSEA plot showing enrichment of HALLMARK_TGFβ_signalling gene set (M5896) in β6 induced versus control 1089^iβ6^ cells. Data are representative of two independent experiments. NES normalised enrichment score. **b** Expression of β6, phospho-, total SMAD2/3 and total SMAD4 upon doxycycline (1 µg/mL) treatment in 1089^iβ6^ cells grown in 2D for 48 h. Blots are representative of two independent experiments. **ci** Immunofluorescence staining of DAPI (blue) and SMAD4 (pink) and (**cii**) quantification of nuclear SMAD4 intensity in 1089^iβ6^ cells treated with treatment for 48 h, where each dot represents one cell. Scale bar = 100 µm (or 50 µm for inserts). ****p < 0.0001 (Two tailed *T* test). **d** Relative luminescence in 1089^iβ6^ cells co-transfected with SMAD-SBE firefly and renilla firefly reporter constructs, treated with 48 h doxycycline (1 µg/mL), SB431542 (10 µM) and/or stimulated with TGFβ (5 ng/mL). **e** MMP13 mRNA expression in 1089^iβ6^ cells treated doxycycline (1 µg/mL) and SB431542 (10 µM) for 48 h. *p < 0.05, **p < 0.01 (One-way ANOVA with multiple comparisons). Data are presented as mean ± SEM, with biological replicates indicated by different colours. Data are representative of three independent experiments.

Having determined β6**-**induced MMP13 as a driver of invasion, we next sought to investigate the mechanism by which β6 expression was driving MMP13 upregulation. As well-characterised ligands of αvβ6 include the Transforming Growth Factor Beta (TGFβ) pro-peptide^42–44^, we hypothesised an increase in TGFβ signalling as a consequence of β6 expression. Gene set enrichment analysis (GSEA) of RNAseq data suggested an enrichment of the TGFβ signalling signature upon β6 induction in 1089^iβ6^ and Myo^iβ6^ cells grown in 2D (Fig. 4a, Supplementary Fig. 4a, b). Indeed, increased basal canonical TGFβ signalling was confirmed by western blot analysis, revealing enhanced phosphorylation of SMAD2/3 following β6 induction in 1089^iβ6^ cells (Fig. 4b). Although total SMAD4 expression remained unchanged following β6 induction, an increase in its nuclear localisation was observed by immunofluorescence, suggesting increased SMAD4-mediated transcription (Fig. 4c). This was confirmed using a SMAD-SMAD binding element (SBE) luciferase reporter assay, which revealed an increase in SMAD driven transcription following β6 induction, both with and without TGFβ stimulation (Fig. 4d). Treatment of β6-induced 1089^iβ6^ cells with TGFβR inhibitor (SB431542; 10 µM) for 48 h blocked transcriptional upregulation of MMP13, indicating that β6-driven MMP13 upregulation occurs through a TGFβ-dependent mechanism (Fig. 4e). To confirm the role of TGFβ signalling in facilitating β6-induced invasion in a 3D context, HB2/1089^iβ6^ spheres were treated with SB431542 and the effect on invasion was assessed. β6-induced spheres showed reduced invasion following TGFβR inhibition, confirming the requirement for β6-driven TGFβ signalling in facilitating β6-induced invasion (Supplementary Fig. 4c).

### MMP13 expression requires β6/ TGFβ-mediated epigenetic regulation

**Fig. 5:**
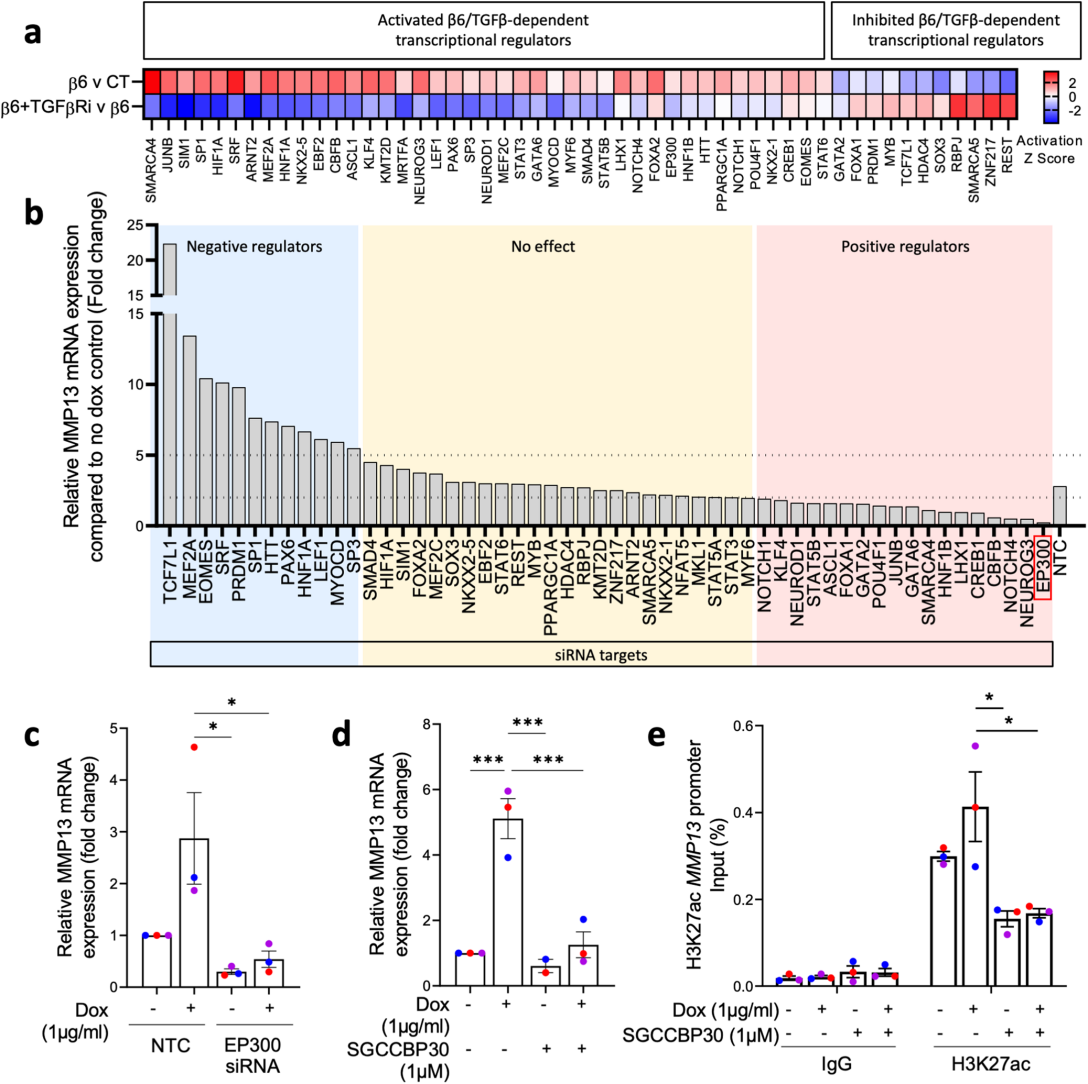
β6 drives TGFβ dependent activation of EP300, which in turn regulates MMP13 expression. **a** Predicted activation Z-scores of TGFβ dependent β6-driven transcriptional regulators, based on expression changes of target genes in 1089^iβ6^ cells following β6 induction. **b** Relative MMP13 mRNA expression following siRNA knock down of transcriptional regulators. NTC non-targeting control. Fold change <2 indicates positive regulators of MMP13 (red) and fold change >5 indicates negative regulators of MMP13 (blue). **c** Relative MMP13 mRNA expression in 1089^iβ6^ cells transfected with NTC or EP300 siRNA, treated with doxycycline (1 µg/mL) for 48 h. **d** Relative MMP13 mRNA expression in 1089^iβ6^ cells treated with doxycycline (1 µg/mL) and/or SGCCBP30 (1 µM) for 48 h. **e** ChIP-qPCR analysis for enrichment of H3K27ac at the *MMP13* promoter region in 1089^iβ6^ cells following treatment with doxycycline (1 µg/mL) and/or SGCCBP30 (1 µM) for 48 h. *p < 0.05, ***p < 0.001 (One-way ANOVA with multiple comparisons). Data are presented as mean ± SEM with biological replicates indicated by different colours. Data are representative of three independent experiments.

After identifying β6-driven MMP13 upregulation to be TGFβ-dependent, we next investigated direct transcriptional regulators downstream of TGFβ signalling that could regulate MMP13 expression. Given our finding that MMP13 expression was regulated independently of SMAD4 (Supplementary Fig. 5a), we used Ingenuity Pathway Analysis to predict upstream regulators of β6-driven, TGFβ-dependent, transcriptional regulators determined from our 1089^iβ6^ RNAseq data. This approach identified 54 TGFβ-dependent hits, with 43 β6-activated and 11 β6-inhibited transcriptional regulators (Fig. 5a). To identify MMP13-specific regulators, each of the 54 hits was individually knocked down by siRNA, and the impact on β6-driven MMP13 transcription was assessed. As β6-induced MMP13 mRNA upregulation typically fell between 2-5-fold in non-targeting control siRNA (NTC) conditions, knockdown of regulators that resulted in below 2-, or above 5- fold, were classed as positive and negative regulators, respectively (Fig. 5b). This approach identified the histone acetyl transferase EP300 as a potential MMP13 regulator, since its knockdown blocked β6-driven MMP13 expression significantly (Fig. 5c). These data were recapitulated following treatment with the dual CREBBP/EP300 inhibitor, SGCCBP30 (1 μM), which yielded a similar loss in MMP13 upregulation following β6 induction, further implicating activation of EP300 as a requirement for β6-induced MMP13 expression (Fig. 5d).

To confirm the regulation of *MMP13* by EP300, chromatin immunoprecipitation (ChIP) was performed to analyse for enrichment of H3K27ac, an epigenetic histone acetylation mark associated with transcriptional activation, at the *MMP13* promoter region following SGCCBP30 treatment. ChIP-qPCR analyses using primer pairs specific for the *MMP13* promoter revealed an enrichment of H3K27ac following β6-induction, which was diminished with SGCCBP30 treatment (Fig. 5e). To confirm the role of EP300 as a driver of invasion in 3D, we next examined whether loss of myoepithelial EP300 in the HB2/1089^iβ6^ sphere model was sufficient to reduce invasion. Consistent with EP300 as a regulator of β6-driven MMP13 expression, EP300 siRNA-treated spheres displayed reduced invasion compared to NTC counterparts, characterised by a significant decrease in the percentage of invasive area despite β6 induction (Supplementary Fig. 5b, c). Taken together, these data suggest that in myoepithelial cells, β6 expression drives TGFβ-dependent activation of EP300 to regulate expression of MMP13 and facilitate invasion.

In line with its potential role in DCIS, data from publicly available invasive breast carcinoma repositories showed that while high RNA expression of β6 in the bulk tumour was associated with higher levels of EP300, MMP13 expression was also significantly increased in high EP300 expressing patients (Supplementary Fig. 5d). Survival analysis revealed that patients with tumours expressing higher protein levels of EP300 demonstrated poorer overall survival, with a hazard ratio of 3.54, further supporting that EP300 may also play a role in facilitating the later stages of breast cancer progression (Supplementary Fig. 5e)^45^.

### β6 integrin-driven invasion is EP300/MMP13 dependent in a physiomimetic ductal model of DCIS

**Fig. 6:**
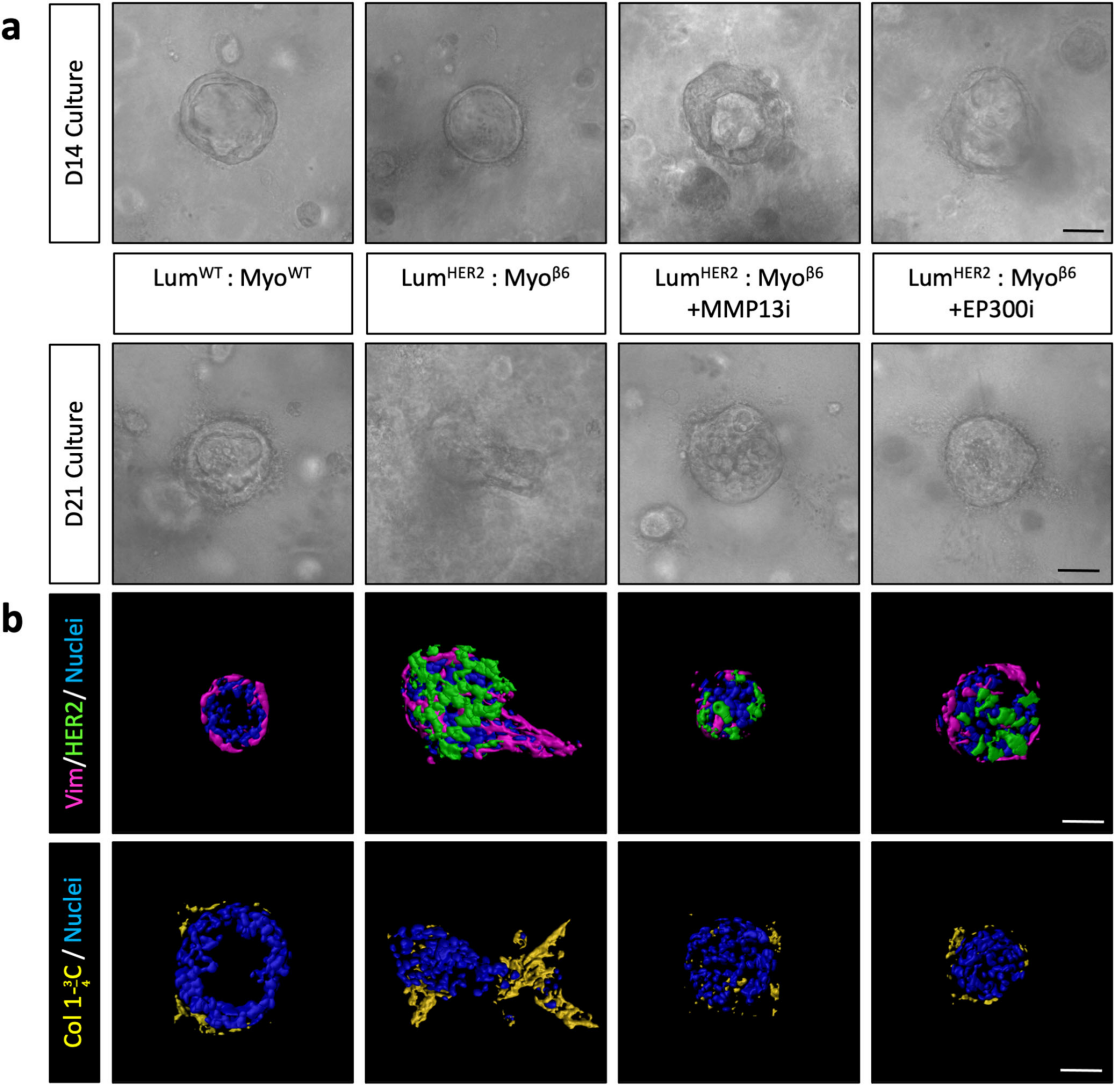
β6 integrin-driven invasion is EP300/MMP13 dependent in a physiologically relevant ductal model of DCIS. Primary luminal and myoepithelial cell ductal structures are formed 14 days after embedding into collagen gels and subsequently treated with doxycycline (1 µg/mL) for a further 7 days to induce transgene expression. To inhibit activity of MMP13 and EP300, ducts were treated with MMP13i (CAS-544678 85-5 1 µM) and EP300i (SGCCBP30 1 µM), respectively. **a** Representative light micrographs of ductal structure at 14 days post embedding. **b** Imaris reconstructions of luminal/myoepithelial ductal structures after 21 days of culture with HER2 luminal expression −/+ myoepithelial β6 expression and inhibition of MMP13 or EP300 where Nuclei (blue), Vimentin (magenta), HER2 (green) and Cleaved Collagen (yellow). Scale bar = 20 µm.

Having shown that loss of the myoepithelial EP300/MMP13 axis blocked invasion in β6 integrin-expressing spheroid models, we recapitulated this in our ductal model of DCIS (Fig. 6, Supplementary Fig. 6), where primary luminal and myoepithelial cells from reduction mammoplasty patients are recombined in Collagen-I to form breast ducts possessing an intact layer of myoepithelial cells^31^. Co-expression of luminal HER2 and myoepithelial β6 integrin was induced upon the formation of ductal structures after 14 days of culture, and the effects of MMP13 and EP300 inhibition on invasion were assessed (Fig. 6a, b).

As expected, deposition of a correctly polarised basement membrane was consistent across conditions, as highlighted by Laminin deposition (Supplementary Fig. 6). Cleaved Collagen-I analysis was consistent with findings observed in the HB2/1089^iβ6^ spheroid model, displaying increased Collagen proteolysis surrounding myoepithelial-led protrusions (Fig. 6b). As with our spheroid model, inhibition of MMP13 reduced Collagen proteolysis as well as myoepithelial-led invasion into the surrounding matrix, confirming MMP13 as an active driver of β6-dependent invasion (Fig. 6a, b). Targeting of EP300 was also sufficient to reduce both invasion and Collagen-I cleavage, highlighting EP300 as a critical regulator of MMP13 transcription in this model (Fig. 6a, b).

### Myoepithelial MMP13 is associated with β6 expression in DCIS

**Fig. 7:**
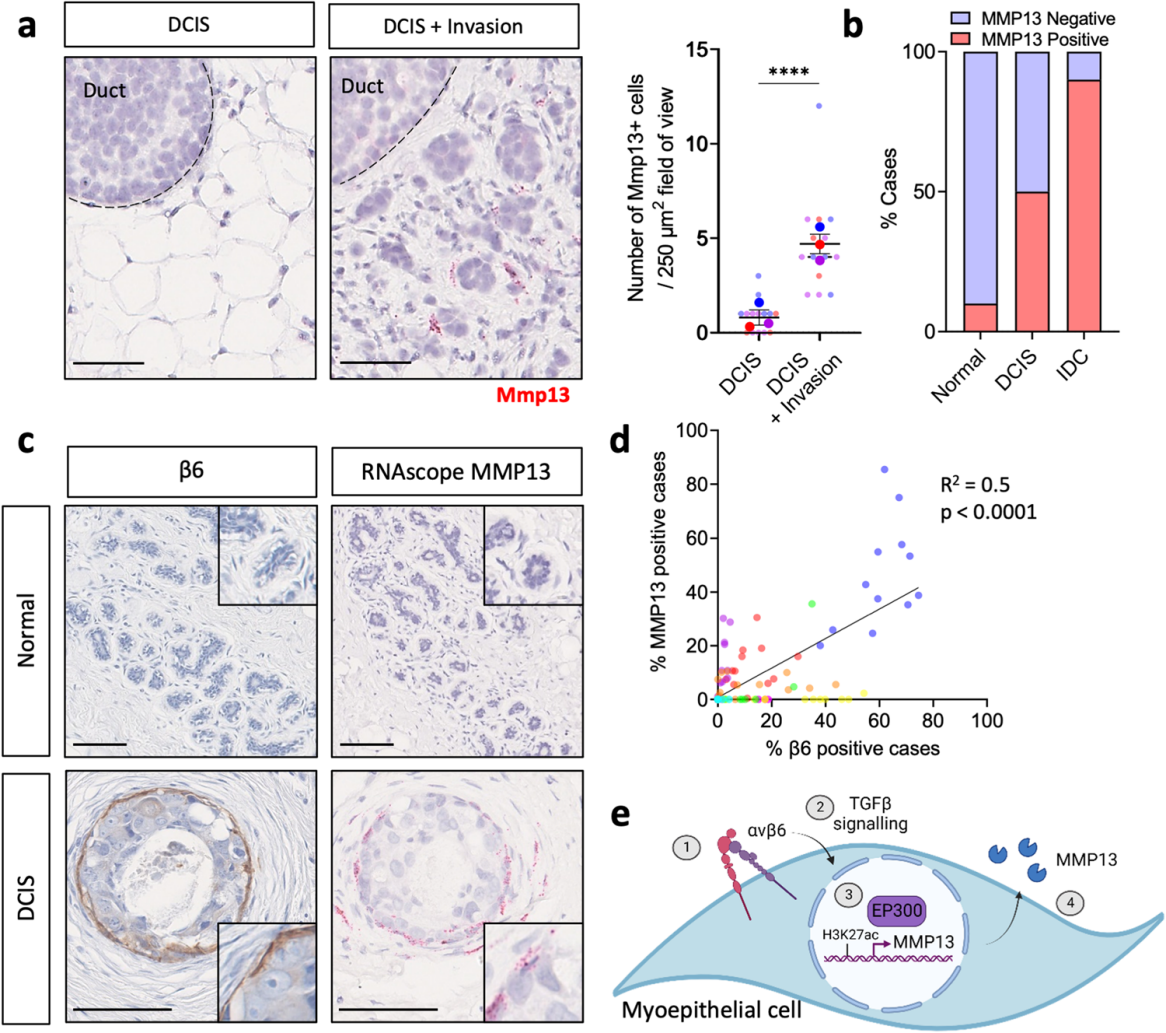
MMP13 expression is associated with invasion in a mouse model of DCIS and in human samples. **a** Representative Mmp13 RNAscope images of non-invasive and invasive fronts from MCF7-MIND mice at 12 weeks post intraductal injection. Mmp13 expression is indicated by presence of red dots. Graph shows number of Mmp13^+^ cells per 250 µm field of view. *n* = 3 mice. Scale bar = 50 µm. **b** Summary graph showing percentage of MMP13 positive patients across normal (*n* = 10), DCIS (*n* = 10) and IDC (*n* = 10) cases. **c** Representative images of β6 IHC (brown) staining and MMP13 RNAscope (red) detection in normal and DCIS patients. Scale bar = 100 µm. **d** Graph showing percentage of MMP13 and β6 positive cells/per duct across DCIS patients. Data are presented as individual points where each dot represents one duct, with different patients represented by different colours (12 DCIS ducts quantified per patient) (Simple linear regression). **e** Schematic showing proposed mechanism where myoepithelial β6 expression drives TGFβ-dependent activation of EP300 to regulate MMP13 expression.

To investigate our findings in an in vivo setting, we next used the mouse intraductal (MIND) model, where human MCF7 cancer cells are xenografted into the mammary ductal tree. In this model, tumours develop with a greater involvement of the tumour microenvironment and demonstrate features more consistent with clinical breast cancer than traditional fat pad xenografts^46,47^. To detect mouse Mmp13 in the microenvironment, while avoiding detection of cancer cell derived MMP13, mouse-specific Mmp13 probes were used for RNA scope analysis. Interestingly, stromal Mmp13 expression was localised to the periphery of collectively invading cancer cells, compared to non-invasive areas of DCIS where Mmp13 expression was significantly reduced (Fig. 7a).

To investigate myoepithelial MMP13 expression in the context of β6 positive DCIS, a previously established marker of high-risk DCIS^24^, 30 human breast samples, comprising 10 normal breast tissue, 10 DCIS and 10 IDC were analysed for expression of MMP13 by RNAscope. Strikingly, whereas 90% of normal samples were MMP13 negative (9 out of 10), MMP13 was detected in the myoepithelial and fibroblast cells of 50% (5 out of 10) of DCIS cases (Fig. 7b, c). Notably, MMP13 positive cases were also high-grade DCIS, while MMP13 negative cases tended to be intermediate or low grade DCIS (Supplementary Fig. 7a).

In MMP13 positive cases, MMP13 expression was absent from the cancer cells and primarily localised to β6 positive myoepithelial cells, as well as fibroblast cells in the tumour periphery, with a positive correlation observed between the percentage of MMP13 and β6 positive cells per duct (Fig. 7d). In IDC, expression of MMP13 was observed in 90% (9 out of 10) of cases, with expression detected in fibroblasts surrounding areas of invasion (Fig. 7b, Supplementary Fig. 7a). While β6 expression was mostly absent in the cancer cell compartment of DCIS, cancer cells in IDC displayed increased β6 expression (Supplementary Fig. 7a), congruent with findings in a study of β6 in breast cancer^48^. This suggests that once the initial stages of invasion have occurred, cancer cells and fibroblasts may upregulate β6 and MMP13, respectively, to support invasion at later stages of disease^48–50^. Supporting this, patient data from the breast invasive carcinoma TCGA PanCancer atlas data set, revealed a significant association between high β6 and high MMP13 RNA expressing patients (Supplementary Fig. 7b)^51^. Taken together, these data suggest a progressive increase in MMP13 activity from normal breast tissue to DCIS to IDC, implicating a role for β6-driven MMP13 in facilitating invasion.

## Discussion

As a non-obligate precursor lesion, it is suggested that only half of DCIS cases will progress to invasive disease^2,3^. Despite this, virtually all cases are treated with surgery, raising concerns surrounding the overdiagnosis and overtreatment of many women^6^. A lack of genetic and transcriptomic differences between neoplastic cells in DCIS and IDC^9^ has driven focus onto the role of the microenvironment, in an attempt to identify markers predicting breast cancer progression. Interestingly, spatial analysis of breast cancer progression revealed that one of the most influential features in predicting invasive recurrence was related to the myoepithelium rather than the neoplastic cells themselves^26^, suggesting a role for DCIS-associated myoepithelial cells in promoting disease progression

Epithelial β6 integrin has been implicated as a driver of progression in cancer, with its expression associated with an aggressive tumour phenotype and reduced survival^48,52–56^. However, these studies mostly describe an upregulation of β6 in cancer cells, opposed to expression in other epithelial derived cells of the tumour microenvironment, such as myoepithelial cells. Here, we present a 3D spheroid model incorporating β6-inducible myoepithelial cells with breast luminal cells in a physiologically relevant matrix, enabling the myoepithelial mechanisms that underpin early-stage breast cancer progression to be dissected. 3D models of the tumour microenvironment are an excellent tool to dissect cellular crosstalk and are becoming a key component of a researcher’s toolkit^57^. Our heterocellular model system offers a unique advantage over others, such as the MCF10A cell line model, which has the capacity to form spheroids but tends to adopt a mixture of both luminal and myoepithelial characteristics^58,59^. Critically, our model allows for the manipulation of distinct myoepithelial and luminal compartments, while maintaining their cellular phenotypes, to recapitulate behaviour seen in human breast tumours. We show that upregulation of the high-risk DCIS marker β6 integrin, in the myoepithelial compartment, drives myoepithelial-led invasion of luminal cells in our spheroid models. As luminal cells are unable to invade in the absence of a myoepithelial conduit (Supplementary Fig. 1f), this suggests that during the early stages of invasion, β6-expressing myoepithelial cells may adopt characteristics similar to those of cancer-associated fibroblasts. This phenomenon is frequently observed during stromal-led invasion, where invading cancer cells maintain their epithelial phenotype and exploit non-malignant cells of the microenvironment to pave the way for invasion^34,60^. Interestingly, in a model of murine breast cancer invasion, leading cells have been shown to adopt the basal CK14 + characteristics of myoepithelial cells^35^. However, as our model incorporates a myoepithelial cell compartment, this provides evidence that cancer cells can rely on resident myoepithelial cells, and thus maintain their luminal state during invasion. A key advantage of our hanging drop spheroid approach is its tractability, but it lacks the intact myoepithelium present in our primary cell model of ductal bilayer development^31^. Therefore, we importantly confirmed that all our key findings were recapitulated in this approach, where an intact myoepithelium is present from 14 days of culture.

MMP13 has previously been implicated as a marker in the transition of DCIS to IDC, where its expression has been localised to stromal myofibroblasts in areas of microinvasion^50^. However, the functional relevance of myoepithelial MMP13 as an active driver of disease progression has yet to be shown. Here, we identify MMP13 as a key protease in facilitating the progression of early stage breast cancer, with significantly reduced invasion observed upon inhibition or knockdown of myoepithelial-MMP13 across 3D models. These findings were corroborated in clinical DCIS samples, where MMP13 expression was present in β6 positive myoepithelial cells. MMP13 expression was increased further in IDC cases, with MMP13 expression detected in both myofibroblasts and cancer cells. This implies that once the initial stages of invasion have occurred and the β6/MMP13 expressing-myoepithelial cell layer is lost, cancer cells may upregulate β6 and MMP13 expression to facilitate their invasion, alongside MMP13 positive myofibroblasts. This is supported by studies demonstrating upregulation of β6 in cancer cells in IDC^48^, as well as an upregulation of MMP13 in fibroblasts^49,50,61^, and can be further reinforced by data from the breast invasive carcinoma TCGA cohort, where we identify a positive correlation between expression of β6 and MMP13^51^. Despite the robust effect of MMP13 knockdown on invasion in our models, we appreciate that several other myoepithelial metzincins may also be involved in promoting the transition of DCIS to IDC^24,62,63^.

We further show that β6-mediated MMP13 upregulation occurs via a TGFβ-dependent mechanism (Fig. 7e). This is consistent with existing literature where β6 drives the upregulation of several MMPs through TGFβ signalling^35^. Despite this, the tumour-promoting role of TGFβ is often observed during later stages of disease in cancer cells, following mutation or allelic loss of pathway components^64^. Here we demonstrate that TGFβ signalling in the myoepithelial compartment may in fact play a role in influencing progression during the early disease. The role for TGFβ influencing progression can be further supported by immunohistochemical analysis, where overexpression of TGFβRs is associated with a worse prognosis in breast cancer^65,66^.

Our data suggest that while β6-driven MMP13 expression in myoepithelial cells is TGFβ dependent, its transcriptional regulation occurs independently of canonical SMAD pathways, as SMAD4 knockdown had negligible effects on MMP13 expression. The most striking result from our β6/TGFβ-dependent transcriptional regulator screen was diminished expression of MMP13 following knockdown of the histone acetyltransferase, EP300. These results are consistent with regulation of MMP13 in osteoblasts during bone remodelling, where EP300 and RUNX2 are required to mediate activation of MMP13 transcription^67,68^. Indeed, our experiments show that in the context of myoepithelial cells, EP300 activity is critical for MMP13 expression, as either knockdown or inhibition were sufficient to block β6-driven MMP13 upregulation. Mining publicly available datasets, we identified that high expression of EP300 in breast cancer patients was associated with reduced and overall survival, further supporting a role for EP300 expression and activity in facilitating disease progression^45^.

Overall, we have shown that myoepithelial cells overexpressing β6 integrin adopt an invasion-promoting phenotype, as demonstrated by an increase in their ability to lead the invasion of luminal cells into the matrix. This is mediated by increased TGFβ signalling, which facilitates epigenetic regulation of MMP13 through EP300 (Fig. 7e). Myoepithelial-derived MMP13 may therefore facilitate DCIS progression, with this mechanism providing a means for further investigation into its role as a marker of high-risk DCIS.

## Methods

### Cell lines and tissue culture

HB2 luminal cells^32^ were a kind gift from Prof Valerie Speirs (University of Aberdeen) and were cultured in DMEM (Gibco), supplemented with 10% foetal bovine serum (FBS) (Gibco), 0.5 μg/mL hydrocortisone (Sigma-Aldrich) and 5 μg/mL Insulin (Sigma-Aldrich). 1089 myoepithelial cells^24^ were cultured in Ham’s F12 medium (Gibco), supplemented with 10% FBS, 0.5 μg/mL hydrocortisone, 10 ng/mL Epidermal Growth Factor (EGF; Sigma-Aldrich) and 5 μg/mL Insulin (Sigma-Aldrich). Primary luminal and myoepithelial cells were isolated as previously described^31,69,70^. In brief, fresh human breast tissues were digested in 1 mg/mL collagenase 1 A and 0.5 mg/mL hyaluronidase and further digested to single-cell suspensions with a 0.05%/0.02% trypsin/EDTA solution (Hyclone) containing 0.4 mg/mL DNAse (Roche Life Science) for 10 min at 37 °C for subsequent cell isolation by fluorescence-activated cell sorting (FACS)^31,69,70^. Primary luminal cells were cultured in DMEM:F12 (Sigma) supplemented with 10% FBS, 0.5 μg/mL hydrocortisone, 10 ng/mL EGF and 5 μg/mL insulin. Primary myoepithelial cells were cultured on 0.08 mg/mL Collagen I (Corning Life Sciences)-coated plates in HuMEC medium (Gibco), supplemented with 0.4% bovine pituitary extract (BPE; Invitrogen), 0.5 μg/mL hydrocortisone, 10 μg/mL apo-transferrin, 10 ng/mL EGF and 5 μg/mL insulin^31,69,70^. All cells were incubated in a 5% CO_2_ humidified atmosphere at 37 °C. Cells were frequently tested for mycoplasma contamination.

### Primary ductal model

Primary ductal cultures were performed as previously described^31^. In brief, luminal and myoepithelial cells were seeded in collagen coated (0.08 mg/mL) 6-well plates, with approximately 1 × 10^5^ cells/well, 24 h prior to lentiviral transduction with constructed HER2 and β6 pINDUCER21 plasmids. 48 h post transduction, luminal and myoepithelial cells were combined in a 1:1 ratio, to give a final concentration of 4.8 × 10^5^ cells/mL. Equal amounts of collagen gel mix, consisting of 4 mg/mL Collagen type I (Corning Life Sciences) and 25 mM HEPES prepared in complete Ham’s F12 medium, adjusted to neutral pH with NaOH, was added to the cell suspension to give a final concentration of 2 mg/mL. Once gels had polymerized and set for 30 min at 37 °C, gels were overlaid with luminal culture medium. Ductal structures formed by 14 days of culture after which cultures were treated with 1 µg/mL doxycycline to induce transgene expression alongside indicated treatments.

### HB2/1089^iβ6^ 3D sphere culture

HB2 and 1089 cells were combined in a 2:1 ratio to a concentration of 3.3. x 10^4^ cells/mL in 0.24% methylcellulose. 20 μL hanging droplets (containing 500 cells/droplet) of the methylcellulose cell suspension was pipetted onto the underside of a 100 mm dish lid and incubated overnight at 37 °C to form spheres. Collected spheres were resuspended in Collagen gels, consisting of 4 mg/mL Collagen type I (Corning Life Sciences) and 25 mM HEPES prepared in complete Ham’s F12 medium, adjusted to neutral pH with NaOH. 50 µL of the sphere-gel mixture, containing approximately 6 spheres, was added per well of a 96-well plate prior to gel polymerisation at 37 °C for 30 min with plate inversion. Gels were overlaid with indicated drug treatments in complete Ham’s F12 medium and incubated for 4 days.

### HB2/Myo^iβ6^ 3D sphere culture

HB2 monoculture spheres were made using the hanging drop method mentioned above. Prior to the resuspension of collected HB2 spheroids, primary myoepithelial cells were first added to the neutralised Collagen gel mixture (see above) prepared in complete HuMEC medium, to give approximately 8000 myoepithelial cells per gel. 50 µL of the sphere and primary myoepithelial cell gel mixture was added per well of a 96-well plate prior to gel polymerisation at 37 °C for 30 min with plate inversion. Gels were overlaid with indicated drug treatments in complete HuMEC medium and incubated for 3 days.

### Sphere quantification and image analysis

To quantify spheres, the percentage of invasive area, the number of projections, the projection length (µm) and the central sphere area (µm^2^) were determined. To determine the percentage of invasive area, the total area of the invading sphere (invading projections + core sphere area) and the core sphere area was quantified. The invasive area was determined by dividing the total area by the core sphere area and multiplied by 100 (Supplementary Fig. 1g). To determine the projection length, the distance from the margin of the core sphere to the leading end of the projection was measured. All sphere quantification was performed using Fiji Software.

### Lentiviral cloning and production

β6 integrin and MMP13-HA overexpression vectors were constructed by subcloning the open reading frame of *ITGB6* and *MMP13* into pINDUCER21 (46948, Addgene) and pLenti CMV Puro DEST (17452, Addgene) respectively, using the Gateway LR Clonase kit (Thermo Fisher Scientific), following the manufacturer’s guidelines. To add a HA-Tag to the C terminus of the MMP13 coding region, the Q5 High-Fidelity DNA Polymerase (New England BioLabs) kit was used with MMP13-HA and AttB primers (Supplementary Table 1).

All lentiviral particles were generated by co-transfecting HEK293T cells with 3.25 μg pCMVR8.2 (12263, Addgene) and 1.7 μg pMD2.G (12259, Addgene) packaging plasmids, and 5 μg of the lentiviral plasmid using FuGENE transfection reagent (Promega), following manufacturer’s guidelines. Virus-containing supernatant was harvested 48 h post transfection and stored at −80 °C. Prior to viral infection, cells were seeded at 5 × 10^4^ cells per well in a 6 well plate and viral supernatant was incubated for 24 h before selection by fluorescence activated cell sorting (FACS) or with antibiotic treatment for 72 h. To fluorescently label cells, H2B-GFP (11680, Addgene) and H2B-RFP (26001, Addgene) constructs were used. For transduction of primary myoepithelial and luminal cells, lentiviral particles were pre-treated with 20 mU/mL neuraminidase (Sigma-Aldrich) at 37 °C for 45 min prior to their addition to cells as previously described^31,71^.

### siRNA transfection

1089 cells were seeded into 6-well plates at a density of 1 × 10^5^ cells/well 24 h prior to transfection, after which cells were transfected with 20 nM pooled siRNA (Horizon Discovery), using Lipofectamine 3000 Reagent (ThermoFisher), according to manufacturer’s instructions. RNA was harvested 72 h post transfection to assess sufficient knockdown by qPCR or western blot as described below.

### Luciferase reporter assay

2 × 10^4^ cells per well of a 24-well plate were co-transfected with 170 ng Renilla luciferase control plasmid (E2231/E2261, pRL, Promega) and 330 ng of reporter plasmid encoding SBE-Firefly luciferase with Lipofectamine LTX/Plus reagent (Invitrogen), following manufacturer’s instructions. 24 h post transfection, cells were treated with indicated drug conditions. For the TGFβ stimulated conditions, cells were serum-starved for 24 h prior to stimulation with 5 ng/mL of TGFβ for 24 h. Activity of Renilla and Firefly reporters was assessed using the Dual-Glo luciferase assay system (E2920, Promega).

### Immunofluorescence

1089 cells cultured on coverslips were fixed in 4% formaldehyde for 10 min at room temp, permeabilised in 0.1% Triton-X100 for 5 min and blocked in 5% bovine serum albumin (BSA) for 30 min, prior to incubation with primary antibody for 1 hr at room temperature (RT). Samples were subsequently incubated with species-appropriate fluorescent secondary antibodies for 1 h at RT, before being mounted using Molecular Probes ProLong Gold Antifade Mountant with DAPI (Invitrogen) (Supplementary Table 2).

For immunofluorescence of spheres, Collagen gels were fixed in 4% formaldehyde for 30 min, permeabilised in 0.1% Triton-X100 for 1 h and blocked in 5% BSA for 1 h, prior to incubation with primary antibody for 48 h in 4 °C with agitation. Gels were then incubated with species-appropriate fluorescent secondary antibodies for 2 h at RT, prior to mounting. Immunofluorescent images were acquired using a LSM710 Zeiss confocal microscope and reconstructed as Z-stacks. All antibody dilutions are listed in Supplementary Table 2. Where indicated, mid sections from confocal z-stack images were reconstructed using IMARIS imaging software (v9.1, Oxford Instruments).

### Western blotting

Cells were lysed with NP-40 lysis buffer (50 mM Tris pH 7.5, 150 mM NaCl, 1% NP40), supplemented with phosphatase and protease inhibitor cocktails (EMD Millipore). Depending on molecular weight, proteins were either separated on 8% SDS-PAGE or 3–8% NuPAGE Tris-Acetate (ThermoFisher) gels, transferred onto a 0.45 µm nitrocellulose membrane (Amersham) and blocked in 5% milk in 0.5% TBS-Tween20 (TBST) prior to incubation with primary antibodies at 4 °C overnight. Membranes were then incubated in species-appropriate horseradish peroxidase (HRP)-conjugated secondary antibodies for 1 h at RT, prior to protein detection using Immobilon Forte Western HRP Substrate (Merck Millipore). All antibodies, as well as corresponding dilutions are listed in Supplementary Table 2. Presented immunoblots within the same figure panel are derived from the same experiment and processed in parallel. Unprocessed immunoblot images are in Supplementary Fig. 8.

### Chromatin immunoprecipitation (ChIP)

The SimpleChIP Plus Sonication Chromatin IP Kit (56383, Cell Signalling) was used, following manufacturer’s guidelines. In brief, after resuspension of the chromatin pellet in ChIP lysis buffer (1% SDS, 10 mM EDTA, 50 mM Tris-HCl pH 8.0 plus 1 X protease inhibitor cocktail), samples were subjected to fragmentation using a Bioruptor pico sonicator (Diagenode) for 5 cycles (30 s ON and 30 s OFF). 5 μg of sonicated, cross-linked chromatin sample per condition was diluted in a 1:4 ratio in 1X ChIP buffer (Cell Signalling) plus 1X protease inhibitor cocktail (Cell Signalling) and subjected to immunoprecipitation with 2.5 μg H3K27ac antibody (Abcam) overnight at 4 °C with rotation. 30 μl of ChIP-Grade Protein G Magnetic Beads (Cell Signalling) were then added to each IP reaction and incubated for 2 h at 4 °C with rotation, prior to a series of low and high salt washes and elution of antibody-protein-DNA complexes in 1X ChIP Elution Buffer. Enriched chromatin samples were then incubated at 65 °C for 2 h with 40 μg Proteinase K (Cell Signalling) to reverse cross-links prior to purification of DNA using the DNA Purification Kit (Cell Signalling) as per manufacturer’s guidelines. DNA was eluted in 50 μL of elution buffer prior to subsequent qPCR using the Luna Universal qPCR Master Mix kit (New England BioLabs).

### Quantitative PCR

RNA was harvested from cells using the Monarch Total RNA Miniprep Kit (T2010S, New England BioLabs), and transcribed using the Luna Script RT SuperMix Kit (3010, New England BioLabs) using 1 μg RNA per reaction, following manufacturers guidelines. For qPCR, 50 ng cDNA and 0.25 μM forward and reverse primers were used per reaction using the Luna Universal qPCR Master Mix (M3003, New England BioLabs), in a Step One Plus Instrument (Applied Biosystems) with cycling conditions as follows: Initial denaturation at 95 °C for 1 min, denaturation at 95 °C for 15 sec, annealing at 60 °C for 15 sec and extension at 72 °C for 30 sec, for 43 cycles. All primer sequences are detailed in Supplementary Table 1. Relative gene expression was calculated using the ΔCt and 2^—ΔΔCt^ method, normalising to reference gene *beta actin*^72^.

### RNA Seq and transcriptional regulator analysis

DESeq2 differential expression analysis was performed on the raw RNA-seq counts of 1089^iβ6^ and Myo^iβ6^ following β6 integrin induction^73^. The resulting matrix was imputed into the GSEA Broad Institute to examine the Hallmark gene set^74^. To perform upstream regulator analysis, QIAGEN Ingenuity Pathway Analysis software was used to determine Activation Z score of predicted transcriptional regulators as described^75^.

### Animals

NOD.Cg-Prkdc^scid^ Il2rgtm^1Wjl^/SzJ (NSG) mice were purchased from Charles River. Animal experiments were performed in accordance with protocols approved by the Service de la Consommation et des Affaires Vétérinaires of Canton de Vaud, Switzerland (VD 1865.5). Mice were maintained and handled according to Swiss guidelines for animal safety with a 12-h-light-12-h-dark cycle in cages enriched with bedding (Aspen Tapvei®), nesting material, cardboard and wood tunnels. Water was acidified (pH between 2.5 to 3) on a resin column (Prominent® CH system) and their diet was supplied by Provimi-Kliba® (3242, irradiated). Housing room temperature was 22 ± 2 °C, with humidity at 55 ± 10%.

### Intraductal xenografts

Three 8 to 12-week-old NSG female mice were anesthetized by intraperitoneal injection with 10 mg/kg xylazine and 75 mg/kg ketamine (Graeub). To prevent eyes from drying while anesthetised, ophthalmic ointment (Viscotears) was applied to the eyes of mice. After shaving and disinfecting the area around the nipple, the intraductal injection was performed by injecting 500,000 MCF7 cells (resuspended in 10 µL of PBS) into the cleaved 3rd and 4th teats with a blunt end Hamilton syringe (HAMI80508, specifications: 50 µL 705 N, gauge 30/13 mm/pst3), as previously described^46^. Paracetamol was added to the drinking water (200 to 300 mg/kg, i.e. 500 mg/250 mL of drinking water) from 1 day prior to the procedure until for 3 days after the intraductal injection. MCF7 cells were grown intraductally for 12 weeks to form mammary tumours. At the end of the period, mice were euthanized by CO_2_ inhalation. Engrafted mammary glands were harvested and fixed in 4% PFA for 2 h, prior to dehydration and paraffin embedding.

### Clinical human tissue

Clinical samples were obtained from surgical specimens from women undergoing breast surgery at Barts Health NHS Trust London. Written consent was obtained and ethical approval was issued by the Cambridge Central Research Ethics Committee (REC) with ethics approval number 21/EE/0072.

### RNAscope

All RNAscope assays were performed following supplier guidelines using the RNAscope 2.5 HD Reagent Kit-RED (322350, ACD, Biotechne) and RNAscope 2.5 HD Detection Reagents-RED (322350, ACD, Biotechne) kits. Following dewaxing, sections were incubated in hydrogen peroxide for 10 min at RT and protease plus reagent for 30 min at 40 °C in a HybEZ oven. RNAscope probes were then applied onto sections for 2 h at 40 °C before incubation with AMP1 (30 min at 40 °C), AMP2 (15 min at 40 °C), AMP3 (30 min at 40 °C), AMP4 (15 min at 40 °C), AMP5 (45 min at RT) and AMP6 (15 min at RT). Slides were then incubated with Fast Red for 10 min at RT and counterstained with Gill’s haematoxylin (Sigma Aldrich). For quantification, scans were uploaded into QuPath software as ‘Brightfield other’ images. Individual ducts were highlighted, and annotations were expanded by 50 µm, with the interior removed to isolate measurements to the myoepithelium and periductal region. Channel 1 was used to detect haematoxylin staining while channel 2 was set to detect red staining (MMP13). Cell segmentation was performed using ‘cell detection’ while chromogen detection was performed using ‘positive cell detection’ with thresholds set according to staining intensity. For DCIS, 12 ducts per patient were annotated and the number of MMP13 positive cells per duct were recorded.

### Immunohistochemistry

Paraffin embedded sections were dewaxed in xylene and rehydrated through an ethanol series, prior to treatment with 2% hydrogen peroxide for 10 min to quench endogenous peroxidase activity. Antigen retrieval was carried out in Pepsin Reagent (Sigma) for 12 min at 37 °C followed by blocking in 4% horse serum in 5% BSA in PBS for 30 min at RT. Sections were subsequently incubated in primary antibody overnight at 4 °C, followed by biotinylated horse anti-mouse secondary antibody (Vectastain) for 30 min at RT. All antibodies, as well as corresponding dilutions, are listed in Supplementary Table 2. Signal was developed using ABC reagent and DAB (Vectastain). Sections were then counterstained with Mayer’s haemotoxylin (Sigma Aldrich) and rehydrated prior to mounting with DPX (Sigma). For quantification, scans were uploaded into QuPath software as ‘Brightfield DAB’ images. Individual ducts were highlighted, and annotations were expanded by 50 µm, with the interior removed to isolate measurements to the myoepithelium and periductal region. Channel 1 was used to detect haematoxylin staining while channel 2 was set to detect DAB staining (β6). Cell segmentation was performed using ‘cell detection’ while chromagen detection was performed using ‘positive cell detection’ with thresholds set according to staining intensity. For DCIS, 12 ducts per patient were annotated and the number of β6 positive cells per duct were recorded.

### Statistical analysis

Depending on normality of data points, statistical significance was determined by two tailed t-test or one-way ANOVA with Mann–Whitney *U* or Kruskall Wallis post-test where appropriate, using Prism (Graphpad Software). *p* < 0.05 was considered significant. Sphere quantification data were presented as superplots, with different colours represented as different biological replicates and averages of each experimental replicate indicated as larger-sized points^76^.

## Supplementary information

Supplementary Figures and Tables

## Data Availability

All RNAseq data generated during the study are publicly available and have been deposited in Gene Expression Omnibus (https://www.ncbi.nlm.nih.gov/geo/) with the accession code GSE224401. Invasive Breast Carcinoma data are available on cBioportal (https://www.cbioportal.org) and were generated by the TCGA research Network (https://www.cancer.gov/tcga). EP300 Kaplan-Meier plots were generated with Kaplan-Meier Plotter (https://kmplot.com) using proteomic data available in the PRIDE Archive – proteomics data repository under the accession number PXD005692. All further datasets generated and analysed in this study are available from the corresponding author upon reasonable request.
