# Supplementary material for: TGFβ-mediated MMP13 secretion drives myoepithelial cell dependent breast cancer progression
Source: NPJ Breast Cancer. 2023 Mar 2;9:9. doi: 10.1038/s41523-023-00513-6 (PMC9981685; doi:10.1038/s41523-023-00513-6)
Supplement: Supplementary file 1 — Supplementary Figures and Tables [file 41523_2023_513_MOESM1_ESM.pdf]

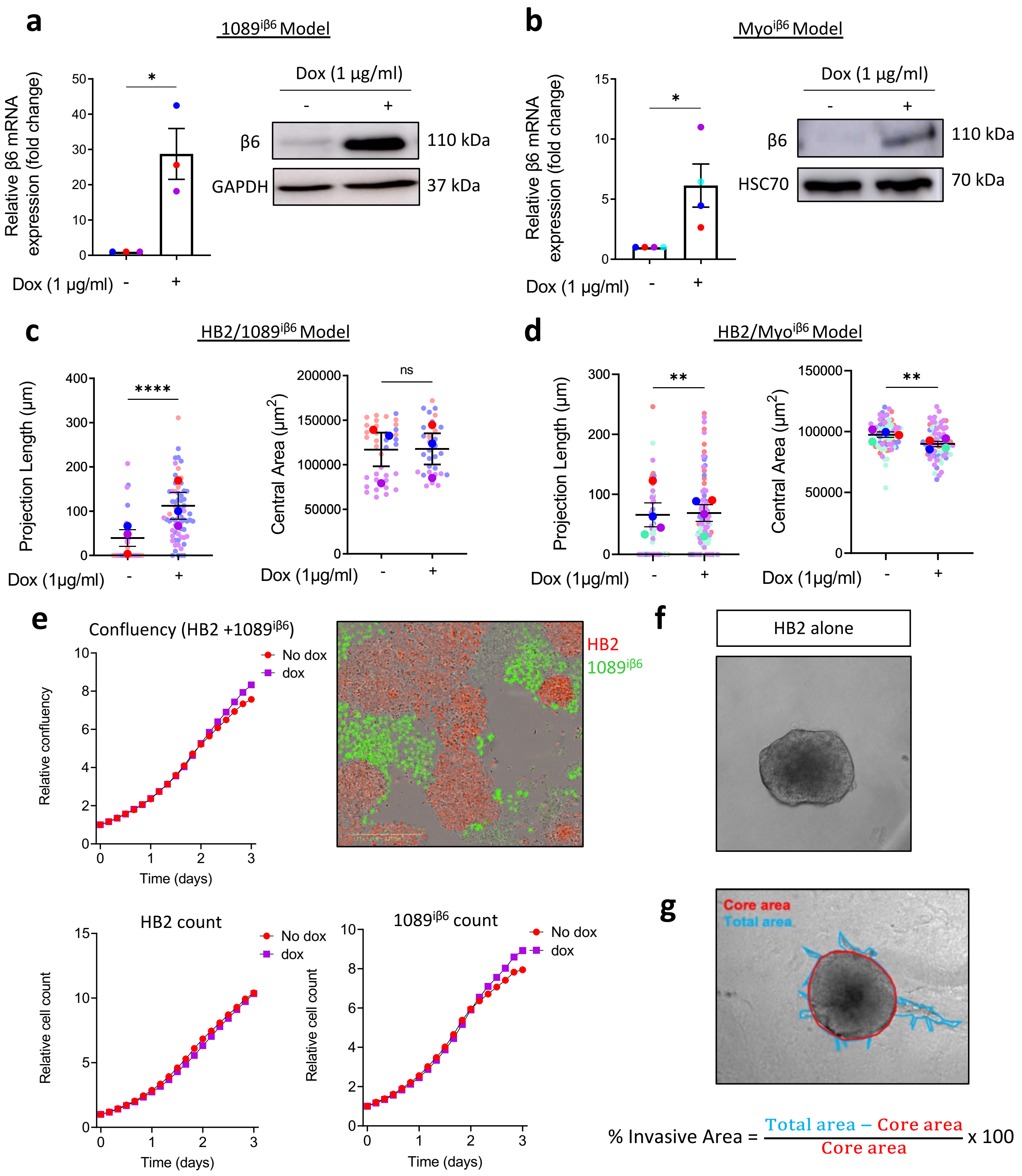

Supplementary Fig. 1: (a-b) Relative β6 mRNA and protein expression following doxycycline (1 µg/mL) in **(a)** 1089iβ6 and **(b)** Myoiβ6cells. *p<0.05 (Two-tailed T test). **(c-d)** Projection length (µm) and central area (µm^2) across treatment conditions in **(c)** HB2/1089iβ6 and **(d)** HB2/Myoiβ6 models 4 days post doxycycline (1 µg/mL) treatment. Data are presented as mean ± SEM where each dot represents one sphere with biological replicates indicated by different colours. Average of biological replicates indicated as larger-sized points. Data are representative of at least three independent experiments. **p<0.01, ****p<0.0001, ns=not significant (Mann Whitney U Test for projection length and two tailed T test for central area). **(e)** Fluorescent HB2 (red) and 1089iβ6 (green) cells were grown in co-culture and treated with doxycycline (1 µg/mL). Growth curves showing relative confluency, HB2 count or 1089iβ6 count over 3 days. **(f)** Representative image of HB2 monoculture sphere 4 days post embedding. **(g)** Calculation of percentage invasive area schematic with equation.

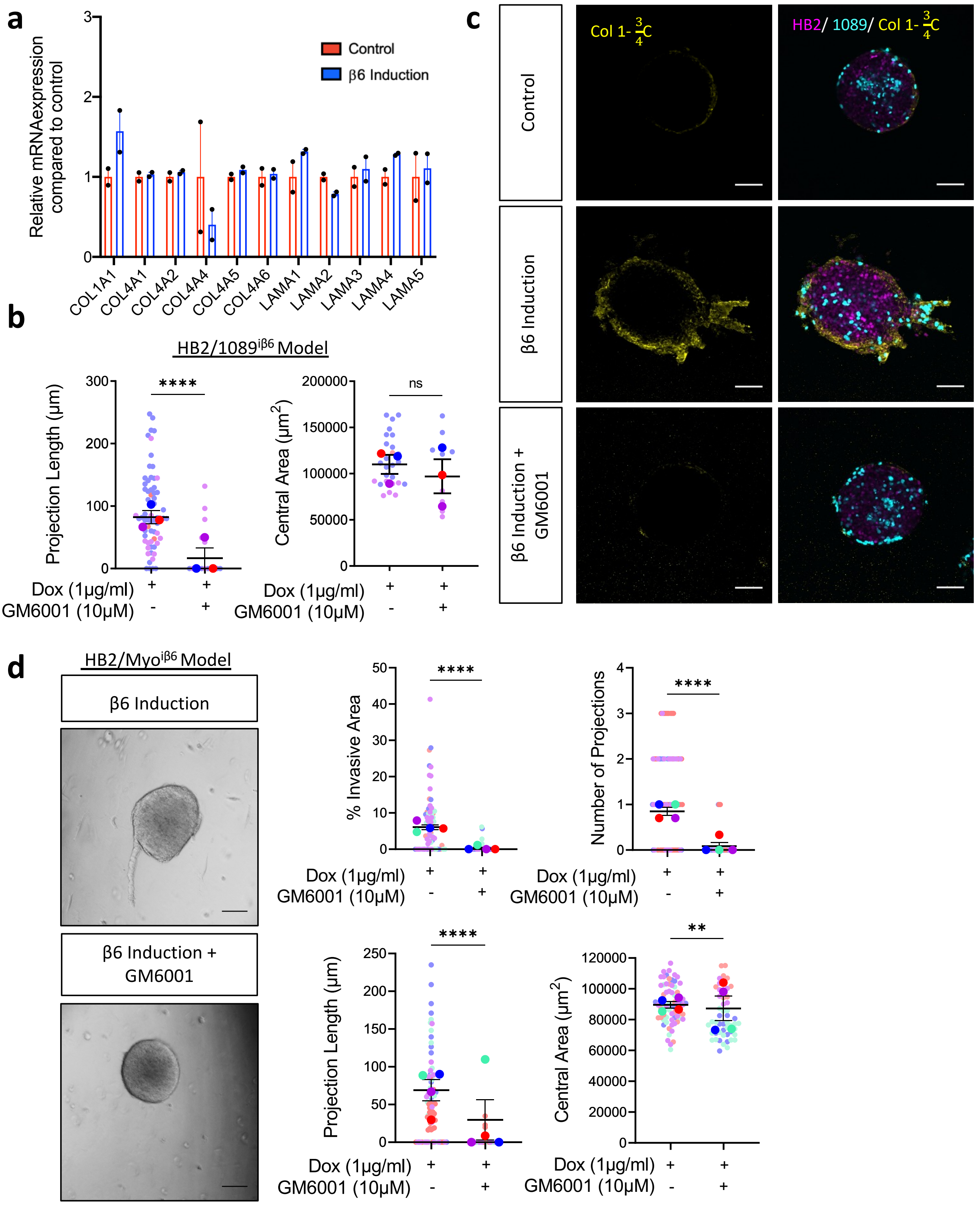

Supplementary Fig. 2: (a) Relative expression of Laminin and Collagen genes compared to control from RNAseq normalised counts. Data are presented as mean ± SEM. Data are representative of two independent experiments. **(b)** Projection length (µm) and central area of spheres (µm2) 4 days post doxycycline (1 µg/mL) and GM6001 (10 µM) treatment in cell line HB2/1089iβ6 sphere model. **(c)** Representative fluorescence images of Cleaved Collagen I (Col 1- 3/4C) in control and β6-induced conditions of HB2/1089iβ6 model 4 days post doxycycline (1 µg/mL) and GM6001 (10 µM) treatment. HB2 (magenta), 1089iβ6 (cyan) and Col 1-3/4C (yellow). **(d)** Representative bright field images of spheres treated with 10 µM GM6001 at day 4 post treatment in HB2/Myoiβ6 primary myoepithelial cell spheroid model and summary graphs showing percentage invasive area, number of projections, projection length (µm) and central area (µm2). Data are presented as mean ± SEM where each dot represents one sphere with biological replicates indicated by different colours. Average of biological replicates indicated as larger-sized points. Data are representative of at least three independent experiments. $^{**}p<0.01$, $^{****}p<0.0001$ (Mann Whitney U Test, with two tailed T test for central area). Scale bar=100 µm.

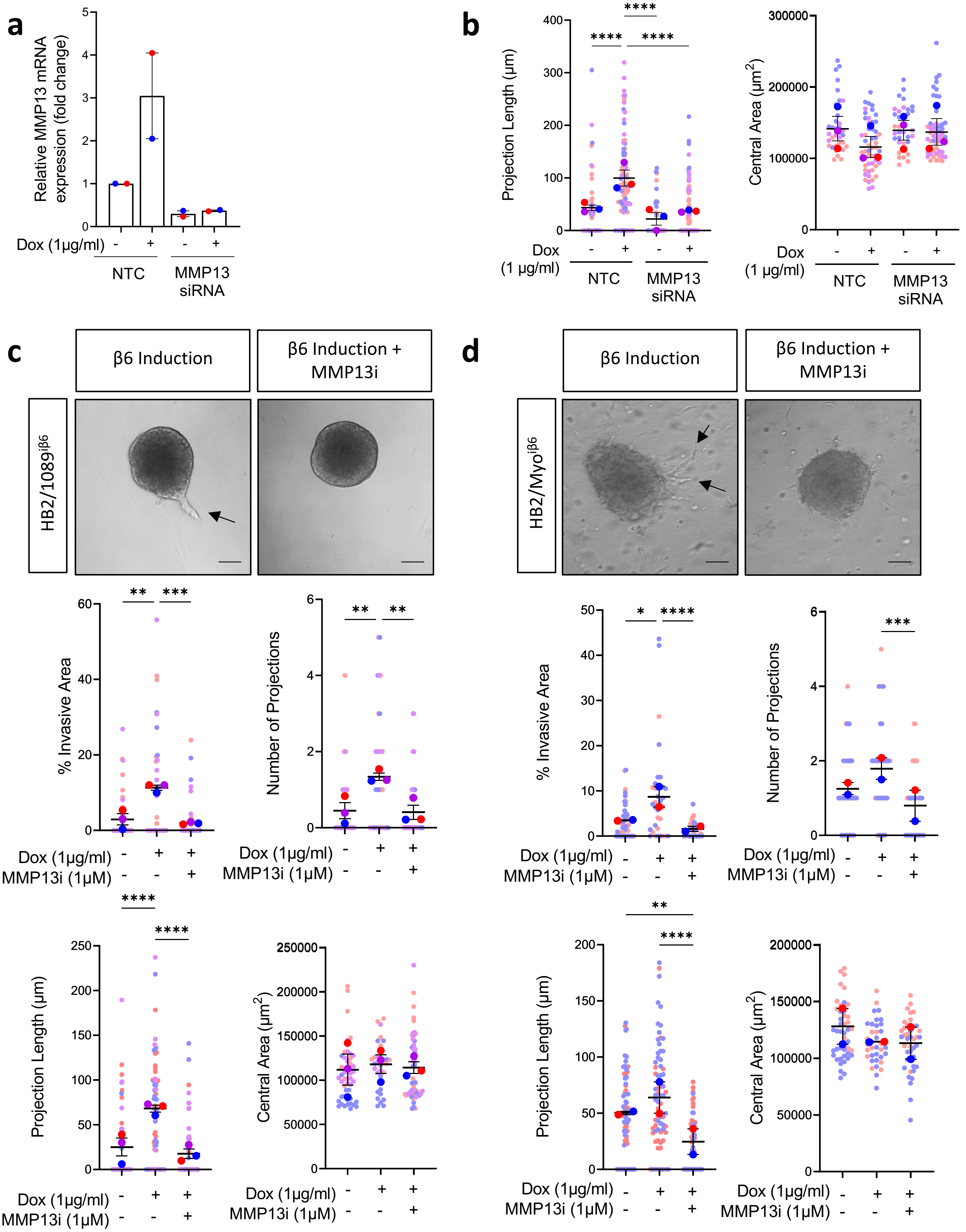

a
Relative MMP13 mRNA expression (fold change)
Dox (1µg/ml)
NTC
MMP13 siRNA
b
Projection Length (µm)
Central Area (µm2)
Dox (1 µg/ml)
NTC
MMP13 siRNA
c
β6 Induction
β6 Induction + MMP13i
HB2/1089iβ6
% Invasive Area
Number of Projections
Projection Length (µm)
Central Area (µm2)
Dox (1µg/ml)
MMP13i (1µM)
d
β6 Induction
β6 Induction + MMP13i
HB2/Myoiβ6
% Invasive Area
Number of Projections
Projection Length (µm)
Central Area (µm2)
Dox (1µg/ml)
MMP13i (1µM)

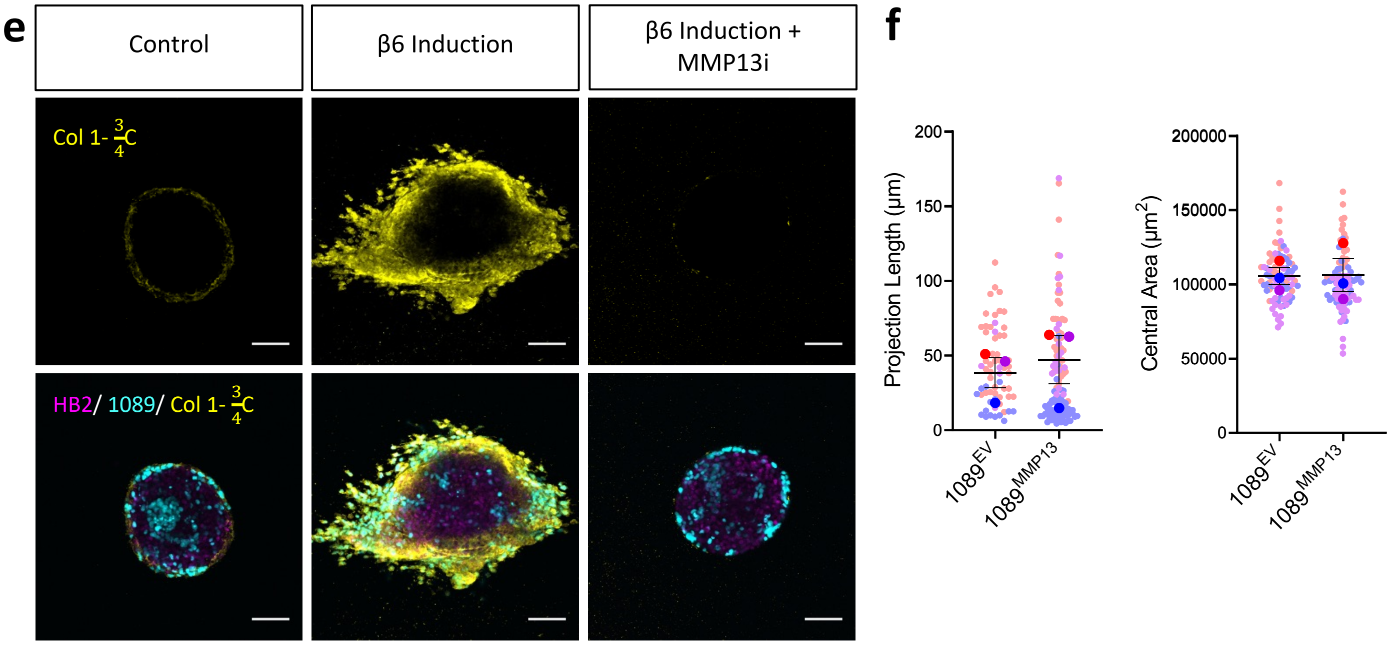

Supplementary Fig. 3: (a) MMP13 mRNA expression in doxycycline (1 μg/mL) treated 1089$^{i\beta6}$ cells 72 hr post transfection with either non-targeting control (NTC) or MMP13 siRNA. Data are representative of two independent experiments. **(b)** Projection length (μm) and central area (μm^{2}) of HB2/1089$^{i\beta6}$ spheres transfected with either non-targeting control (NTC) or MMP13 siRNA after 4 days with doxycycline (1 μg/mL) treatment. **(c-d)** Representative light micrographs at day 4 of doxycycline (1 μg/mL) and CAS-544678 85-5 (MMP13i) (1 μM) treatment in **(c)** HB2/1089$^{i\beta6}$ spheres transfected with either non-targeting control (NTC) or MMP13 siRNA after 4 days with doxycycline (1 μg/mL) treatment. **(c-d)** Representative light micrographs at day 4 of doxycycline (1 μg/mL) and CAS-544678 85-5 (MMP13i) (1 μM) treatment in **(c)** HB2/1089$^{i\beta6}$ and **(d)** HB2/Myo$^{i\beta6}$ sphere models. Summary graphs showing percentage of invasive area**,** number of projections**,** projection length (μm) and central area (μm^{2}). **(e)** Representative fluorescence confocal images of Cleaved Collagen I (Col 1- 3/4C) in control and β6-induced conditions of HB2/1089$^{i\beta6}$ model 4 days post doxycycline (1 μg/mL) and CAS-544678 85-5 (1 μM) treatment. HB2 (magenta), 1089$^{i\beta6}$ (cyan) and Col 1- $\frac{3}{4}$C (yellow). **(f)** Projection length and central area (μm^{2}) across treatment conditions in HB2/1089^{MMP13} spheres. Data are presented as mean ± SEM where each dot represents one sphere with biological replicates indicated by different colours. Average of biological replicates indicated as larger-sized points. Data are representative of three independent experiments. *p<0.05, **p<0.01, ***p<0.001, ****p<0.0001 (Kruskal-Wallis Test or one-way ANOVA, with multiple comparisons). Scale bar=100 μm.

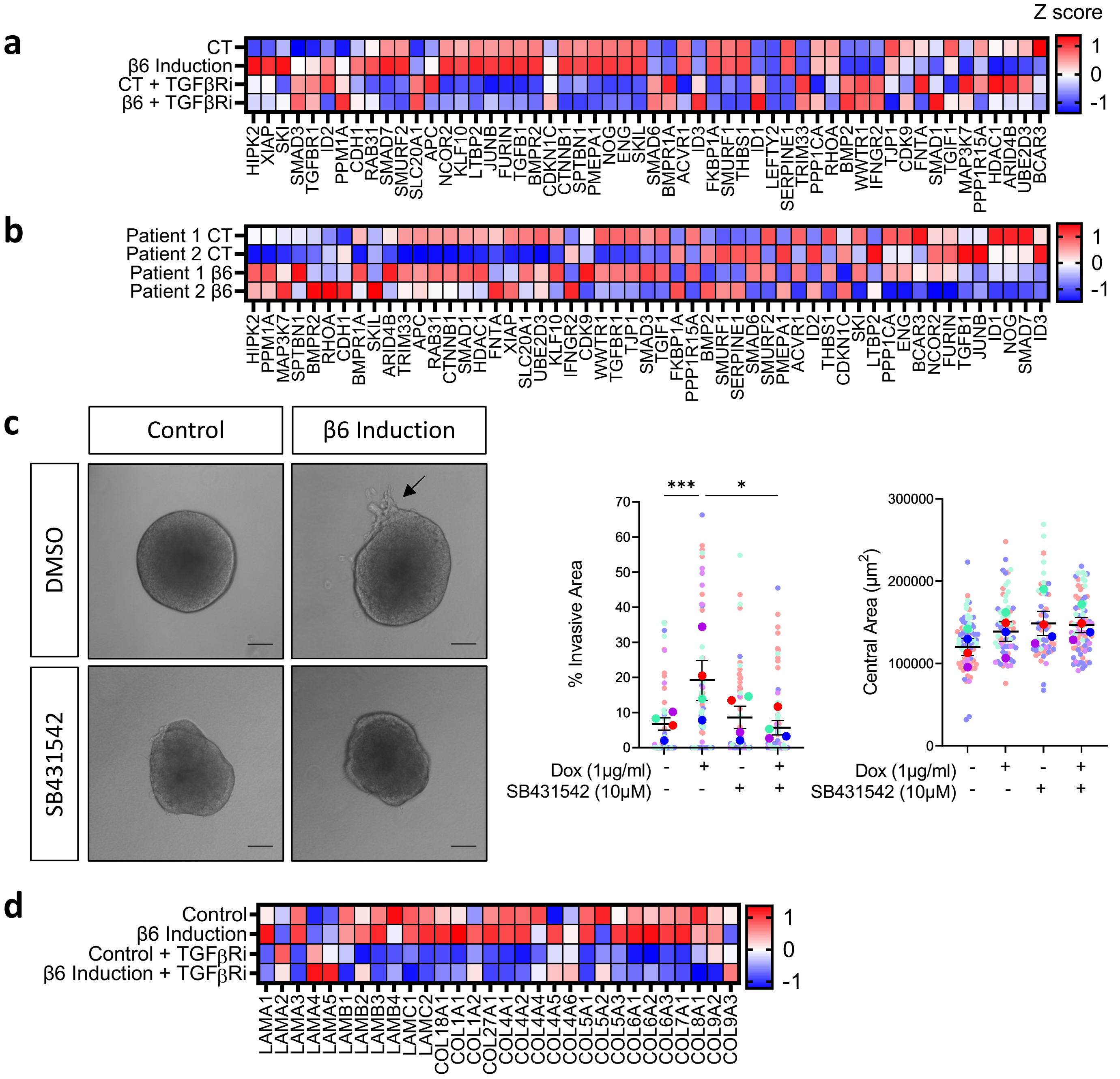

Supplementary Fig. 4: (a-b) Heatmap showing RNA expression (represented as Z scores) of genes from HALLMARK_TGFβ_signalling gene set (M5896) in **(a)** $1089^{i\beta6}$ cells treated with doxycycline (1 μg/mL) to induce β6 expression and/or of SB431542 (10 μM) and **(b)** $Myo^{i\beta6}$ from 2 patients treated with doxycycline (1 μg/mL) to induce β6 expression or 48 hr. Data are representative of two independent experiments. **(c)** Representative light micrographs of HB2/$1089^{i\beta6}$ spheres taken at day 4 of doxycycline (1 μg/mL) and of SB431542 (10 μM) treatment. Scale bar=100 μm. Summary graphs showing percentage of invasive area and central area (μm^2) across conditions. Data are presented as mean ± SEM where each dot represents one sphere with biological replicates indicated by different colours. Average of biological replicates indicated as larger-sized points. Data are representative of four independent experiments. *$p<0.05$, ***$p<0.001$ (Kruskal-Wallis Test with multiple comparisons). **(d)** Expression of Laminin and Collagen (represented as Z scores) in $1089^{i\beta6}$ cells treated with doxycycline (1 μg/mL) to induce β6 expression and/or of SB431542 (10 μM) to inhibit TGFβ signalling. Data are representative of two independent experiments.

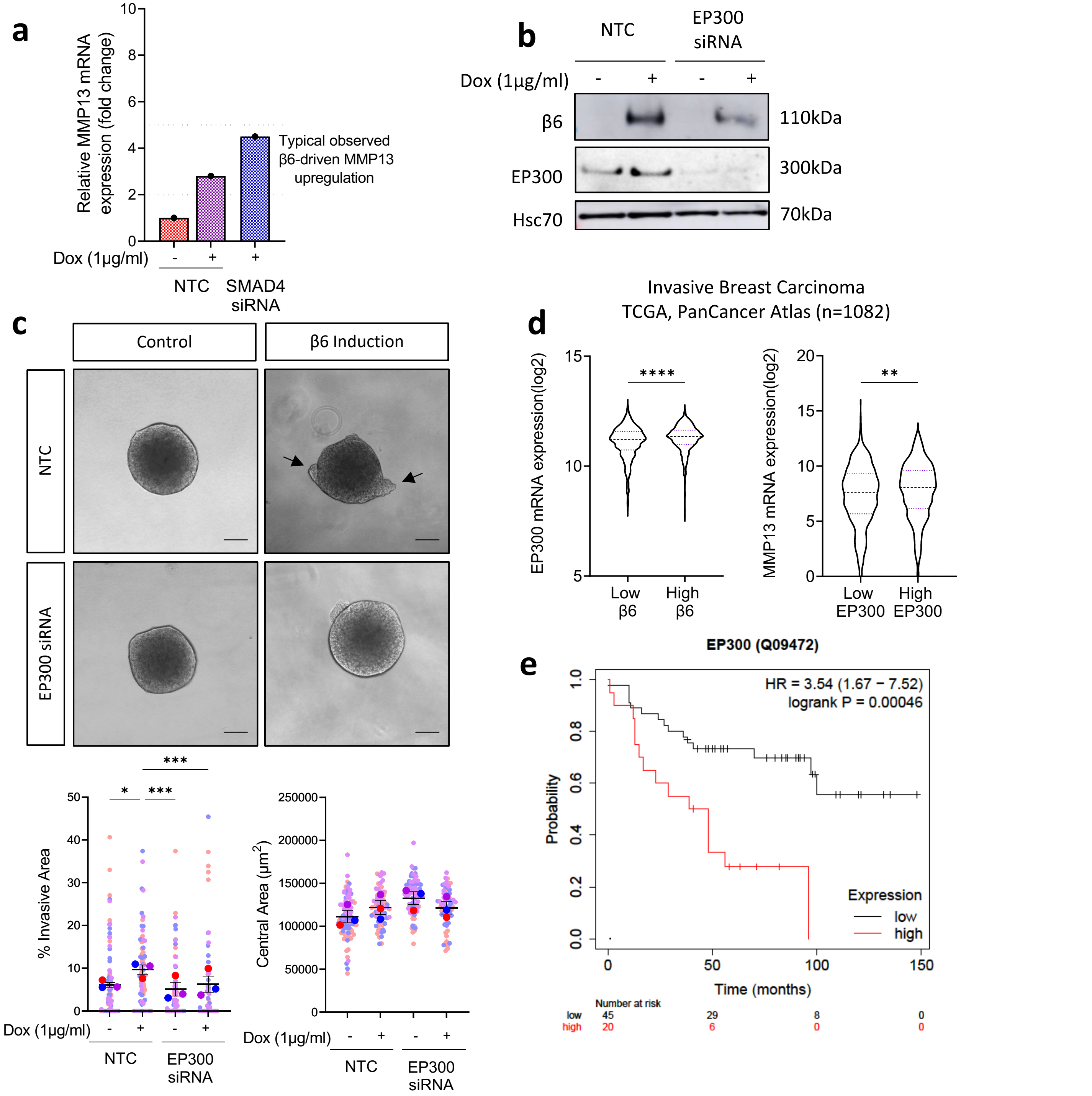

Supplementary Fig. 5: (a) Relative MMP13 mRNA expression following siRNA knock down of SMAD4 in doxycycline (1 μg/mL) treated 1089$^{i\beta6}$ cells compared to non targeting control (NTC). **(b)** Western blot showing β6 and EP300 expression in doxycycline (1 μg/mL) treated 1089$^{i\beta6}$ cells 72 hr post transfection with either NTC or EP300 siRNA. **(c)** Representative light micrographs of HB2/1089$^{i\beta6}$ spheres transfected with either NTC or EP300 siRNA 4 days post doxycycline (1 μg/mL) treatment. Scale bar=100 μm. Summary graphs showing percentage of invasive area and central area (μm^2) across conditions. Data are presented as mean ± SEM where each dot represents one sphere with biological replicates indicated by different colours. Average of biological replicates indicated as larger-sized points. Data are representative of three independent experiments. *p<0.05, ***p<0.001 (Kruskal-Wallis Test with multiple comparisons). **(d)** EP300 and MMP13 mRNA expression in patient samples taken from the breast invasive carcinoma, TCGA, PanCancer Atlas data set. Low or high β6 or EP300 classification was determined based on expression above or below β6 or EP300 median expression. **p<0.01, ****p<0.0001 (Two tailed T test). **(e)** Kaplan Meier plots showing association between EP300 expression and overall survival from the breast cancer data set (Tang *et al*, 2018).

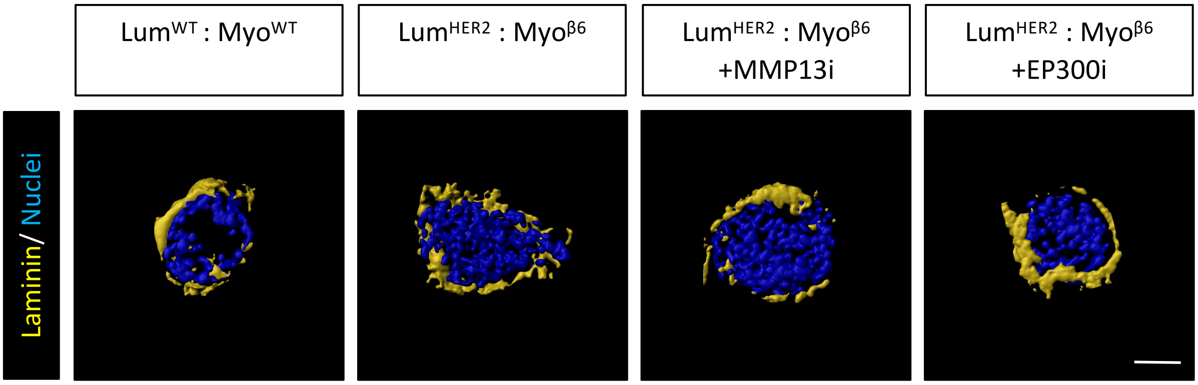

Supplementary Fig. 6: Imaris reconstructions of luminal/myoepithelial ductal structures after 21 days of culture with HER2 luminal expression -/+ myoepithelial β6 expression and inhibition of MMP13 or EP300 where Nuclei (blue) and Laminin (yellow). Scale bar=20 µm.

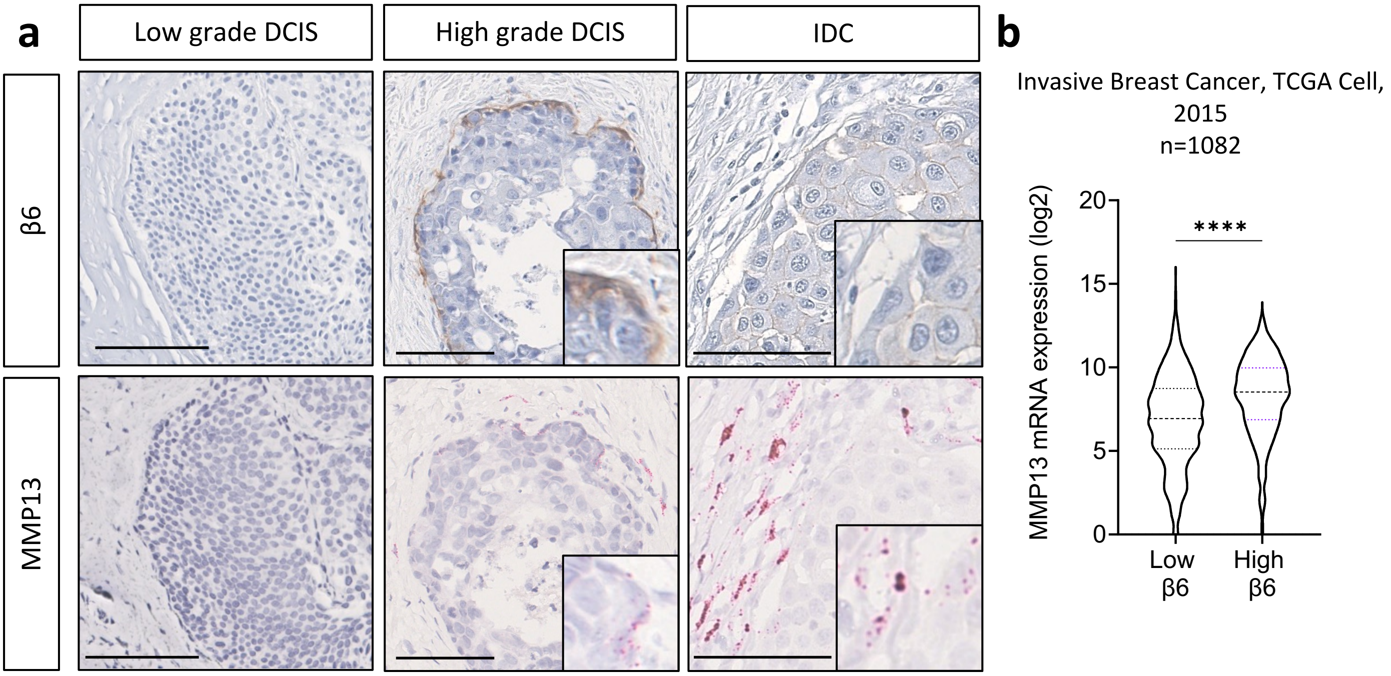

Supplementary Fig. 7: (a) Representative images of β6 IHC (brown) staining and MMP13 RNAscope (red) detection in low grade, high grade and IDC patients. Scale bar=100 µm. **(b)** MMP13 mRNA expression in patient samples taken from the breast invasive carcinoma, TCGA Cell 2015 data set. Low or high β6 classification was determined based on expression above or below median β6 expression. ****p<0.0001 (Two tailed T test).

Unedited blots for Supplementary Fig 1A,B

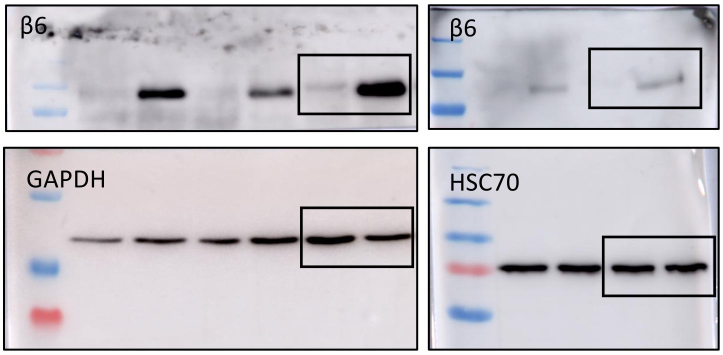

Unedited blots for Supplementary Fig 3C

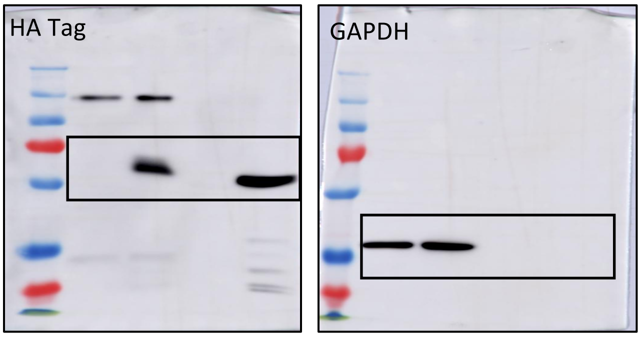

Unedited blots for Supplementary Fig 4B

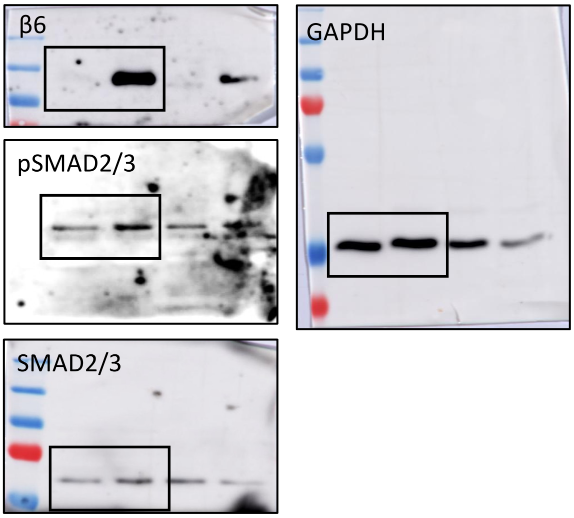

Unedited blots for Supplementary Fig 5B

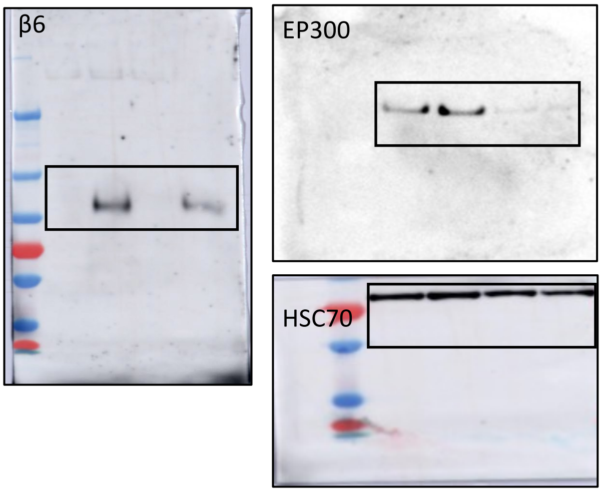

Supplementary Fig. 8: Unprocessed immunoblots related to the indicated blots.

Supplementary table 1. List of primers

Gateway cloning primers		
MMP13-HA	Fw: GGGGACAAGTTTGTACAAAAAAGCAGGCTTC-ATGCATCCAGGGGTCCTGGCTGCC<br>Rv: TTA-AGCGTAATCTGGAACATCGTATGGGTA-CCC-ACACCACAAAATGGAATTTGC	
HA-AttB	Fw: GGGGACAAGTTTGTACAAAAAAGCAGGCTTC-ATGCATCCAGGGGTCCTGGCTGCCT<br>Rv: GGGGACCACTTTGTACAAGAAAGCTGGGTC-TTA-AGCGTAATCTGGAACATCGTATGGGTA	
ITGB6 AttB	Fw: GGGGACAAGTTTGTACAAAAAAGCAGGCTTC-ATGGGGATTGAACTGCTTTGC<br>Rv: GGGGACCACTTTGTACAAGAAAGCTGGGTC-CTAGCAATCTGAGGAAAGGTC	
qPCR primers		Product length
BACT	Fw: AGAGCTACGAGCTGCCTGAC<br>Rv: AGCACTGTGTTGGCGTACAG	184
ITGB6	Fw: AGCCTCTCAGTGTAGGCAGA<br>Rv: GCACCACCTGGTCTCAACTT	91
MMP13	Fw: AACATCCAAAAACGCCAGAC<br>Rv: GGAAGTTCTGGCCAAAATGA	155
ChIP-qPCR primers		
MMP13	Fw: CACAGGCCACTTGAGAGGTT<br>Rv: AAGGTTGGTGGTGAAAGTGAGA	101

Supplementary table 2. List of antibodies and corresponding dilutions

Target	Antibody	Application and dilution
β6	#SC6632 (Santa Cruz)	WB (1:1000)
EP300	#86377 (Cell signalling)	WB (1:1000)
GAPDH	#Mab374 (EMD Millipore)	WB (1:2000)
HA-tag	#3724 (Cell signalling)	WB (1:1000)
HSC70	#SC7298 (Santa Cruz)	WB (1:1000)
pSMAD2/3	#SC11769 (Santa Cruz)	WB (1:250)
SMAD2	#3103 (Cell signalling)	WB (1:1000)
SMAD4	#SC7966 (Santa Cruz)	WB (1:1000) /IF (1:200)
Cleaved Col	#0217-050 (Immunoglobe)	IF (1:100)
Collagen IV	#6586 (Abcam)	IF (1:200)
Laminin	#L9393 (Sigma)	IF (1:200)
β6	#407317 (Calbiochem)	IHC (1:750)
SMA	#M0851 (Dako)	IHC (1:500)
EP300	#275388 (Abcam)	ChIP (5 µg per IP)
